# Low-Carbon Economic Operation of Natural Gas Demand Side Integrating Dynamic Pricing Signals and User Behavior Modeling

**DOI:** 10.3390/e27111120

**Published:** 2025-10-30

**Authors:** Ning Tian, Bilin Shao, Huibin Zeng, Xue Zhao, Wei Zhao

**Affiliations:** 1 School of Management, Xi’an University of Architecture and Technology, Xi’an 710055, China; tning4891@xauat.edu.cn (N.T.); zhaoxue1018@xauat.edu.cn (X.Z.); zhaowei1806@xauat.edu.cn (W.Z.); 2 School of Economics and Management, Chongqing Normal University, Chongqing 401333, China; zenghuibin@xauat.edu.cn

**Keywords:** demand side management, dynamic pricing signals, user behavior modeling, differentiated carbon emission management

## Abstract

Natural gas plays a key role in the low-carbon energy transition due to its clean and efficient characteristics, yet challenges remain in balancing economic efficiency, user behavior, and carbon emission constraints in demand-side scheduling. This study proposes a low-carbon economic operation model for terminal natural gas systems, integrating price elasticity and differentiated user behavior with carbon emission management strategies. To capture diverse demand patterns, dynamic time warping k-medoids clustering is employed, while scheduling optimization is achieved through a multi-objective framework combining NSGA-III, the entropy weight (EW) method, and the VIKOR decision-making approach. Using real-world data from a gas station in Xi’an, simulation results show that the model reduces gas supply costs by 3.45% for residential users and 6.82% for non-residential users, increases user welfare by 4.64% and 88.87%, and decreases carbon emissions by 115.18 kg and 2156.8 kg, respectively. Moreover, non-residential users achieve an additional reduction in carbon trading costs of 183.85 CNY. The findings demonstrate the effectiveness of integrating dynamic price signals, user behavior modeling, and carbon constraints into a unified optimization framework, offering decision support for sustainable and flexible natural gas scheduling.

## 1. Introduction

### 1.1. Background and Motivation

In China, achieving carbon peaking by 2030 and carbon neutrality by 2060 has become a core strategic objective. Under the dual-carbon policy, which guides this transition, the share of low-carbon energy in the energy mix has gradually increased. However, transitioning directly from traditional high-carbon energy sources to renewable energy is clearly not the optimal path, as renewable energy exhibits intermittency and instability. This necessitates further advancements in energy storage systems within the industrial sector [1]. As a low-carbon fossil fuel, natural gas is regarded as a vital transitional bridge from conventional energy to zero-carbon energy sources [2]. At the same time, in natural gas scheduling, ensuring stable and orderly supply has become a critical issue [3]. According to statistics from the National Energy Administration, global natural gas consumption is expected to account for 25% of total energy consumption by 2030, which would be double the level recorded in 2008 [4]. The National Development and Reform Commission of China has also indicated that natural gas will become a dominant primary energy source in the future, with its share expected to reach 15% by 2030 [5]. The use of natural gas to mitigate the intermittency of renewable energy contributes to a more sustainable and environmentally friendly energy supply, thereby accelerating the transition of the energy system toward a cleaner future.

Currently, China’s natural gas industry chain encompasses multiple stages, including upstream exploration and extraction, transportation, storage, liquefied natural gas (LNG) and compressed natural gas (CNG) production, as well as downstream distribution and consumption. Within this vast industry chain, each segment is interconnected and interdependent, working collectively to ensure the effective supply and utilization of natural gas. Within the downstream segment, gas scheduling and distribution at natural gas stations are essential for ensuring system stability and operational safety. These processes directly influence the stability and safety of urban gas scheduling [6]. The scheduling system must strike a balance between demand and supply, ensuring sufficient supply during peak demand periods while also avoiding gas shortages due to inadequate reserves.

Demand response (DR) can guide the flexible load flow within the system through price adjustments or incentive-based rewards, thereby reducing load volatility [7]. In power systems, DR has been widely implemented to smooth the load curve by adjusting users’ consumption behavior. However, the application of DR in natural gas systems (NGS) faces more complex constraints. On one hand, gas transmission and distribution rely on maintaining pressure balance within the pipeline network, requiring the operation of compressors to ensure delivery efficiency. Unlike electricity, natural gas cannot be delivered and consumed instantaneously, and user responses often exhibit significant delays. On the other hand, natural gas scheduling is typically carried out by the dispatch center, which relies on historical consumption data, demand forecasts from key users, reported information, and the terms of contracts signed with users to manage the gas distribution and optimize its scheduling. The absence of a real-time price feedback mechanism limits users’ willingness to adjust gas consumption in the short term. Current natural gas scheduling systems mainly rely on static contractual data rather than dynamic market signals, which constrains the development of flexible and interactive DR mechanisms. Establishing an adaptive price DR mechanism is urgently required to enhance user participation and improve system operational efficiency.

DR still provides a new paradigm for load optimization in NGS [8], but its environmental benefits have not been fully released. With the advancement of the carbon trading market, industrial users of natural gas as a fuel are expected to be incorporated into this market mechanism. Despite being a relatively low-carbon fossil fuel, the extensive utilization of natural gas still results in non-negligible carbon emissions and ecological impacts. Especially in industrial production processes, the high consumption of natural gas can lead to the accumulation of greenhouse gas emissions, thereby affecting long-term goals related to climate change and environmental protection. As a result, integrating natural gas into carbon trading systems will compel users to place greater emphasis on the management and control of carbon emissions [9]. Carbon emissions from natural gas consumption are closely related to combustion efficiency and user type. Existing natural gas demand response models primarily focus on economic benefits [10]. They seldom incorporate carbon emission objectives into the optimization and often overlook the variations in carbon constraints among different user types.

Therefore, this study aims to integrate user response behavior on the demand side with differentiated carbon emission management into the natural gas scheduling system, to promote a shift from passive scheduling to proactive guidance and enhance the economic and environmental performance of NGS under low-carbon constraints.

### 1.2. Literature Review

The optimization of natural gas scheduling has attracted considerable attention in recent research, although the focus of these studies varies. Most existing work concentrates on the operation of CNG main stations or the supply scheduling and reliability analysis of natural gas pipeline networks. Despite growing attention to energy transition, research on downstream urban natural gas systems remains limited, especially concerning low-carbon economic dispatch optimization on the demand side of diverse end users. Liang et al. [11] developed an economic scheduling model for the CNG main station by incorporating a critical peak pricing mechanism. Their results showed that the model effectively reduced electricity operating costs and decreased compressor switching frequency, demonstrating that optimized scheduling can significantly improve operational efficiency and reduce equipment wear. Xiang et al. [10] analyzed the emergency scheduling problem in natural gas pipeline networks (NGPS) and proposed an optimization model for emergency scheduling by modifying the NGPS topology using graph theory methods. The model integrates user weights, satisfaction levels, and reduction factors into the user modeling process, aiming to enhance overall system satisfaction while considering demand-side requirements and operational constraints. Fan et al. [12] proposed a method combining Bayesian networks and deep reinforcement learning to optimize the reliability of natural gas network supply. By integrating probabilistic safety analysis and preventive maintenance, the approach aims to minimize gas shortage risks and reduce maintenance costs.

#### 1.2.1. Demand Side Management in Natural Gas

In energy systems, load shifting is essentially a rescheduling strategy aimed at optimizing resource allocation [13]. Therefore, integrating DR mechanisms into the scheduling process, while accounting for the dynamic changes in user demand, has become a critical approach to enhancing scheduling efficiency, optimizing energy utilization, and promoting sustainable development. In recent years, DR has been widely implemented in power systems, heating systems, and integrated energy systems (IES) [14]. Unlike electricity and heat, which are typically distributed through centralized supply systems, natural gas delivery is highly dependent on complex infrastructure. This introduces unique requirements for the temporal response and regulation mechanisms of DR in NGS. Additionally, DR in natural gas systems may involve delay effects, which mainly arise from the inherent time lag in gas transmission dynamics. Despite these temporal delays, electricity, heating, integrated energy, and NGS can still achieve supply-demand balance through flexible demand adjustment mechanisms, improving operational efficiency and reducing overall costs. The successful integration of DR in the electricity sector and IES offers valuable insights for NGS, particularly in improving demand flexibility and enabling more efficient intelligent scheduling. Basak and Bhattacharyya [15] optimized the cost-effectiveness of microgrids by analyzing the wind speed-to-power conversion model and implementing an economic demand-side management (DSM) strategy. Shi et al. [16] conducted a study on the economic and environmental impacts of integrating DR into IES through simulation. The experimental results demonstrate that the application of DR can significantly reduce both the economic and environmental costs of the system.

Unlike other energy systems, research on DR in natural gas remains relatively limited, resulting in numerous challenges in demand-side optimization, price signal guidance, and carbon emission control. Fan et al. [6] proposed a DR approach based on dynamic pricing, intelligent decision-making, and data-driven forecasting, which successfully achieved peak shaving and valley filling objectives within complex natural gas networks. Zeng et al. [17] developed a dynamic pricing-based natural gas DR model that incorporates own price elasticity and user satisfaction. These studies validate the feasibility of natural gas DR, but they also reveal limitations in capturing cross-price elasticity. Certain limitations also exist in the consideration of compressor costs [18]. To the best of our knowledge, among studies incorporating DR into scheduling systems, only integrated energy systems have addressed the natural gas subsystem.

At present, most studies on downstream natural gas transmission scheduling focus on supply-side constraints, while systematic modeling of demand-side driven optimization strategies guided by dynamic pricing remains insufficient. Existing research often treats price as a static or exogenous variable and assumes fixed price elasticity. The absence of modeling efforts that incorporate time-varying price signals into independent scheduling frameworks restricts the ability to alleviate peak-load pressure. The present study emphasizes the role of flexible demand-side response in optimizing natural gas scheduling. A dynamic time-of-use price elasticity model is introduced on the demand side, enabling price signals to guide and adjust user load, thereby alleviating supply-demand conflicts during peak periods. On the one hand, a more guiding real-time dynamic pricing mechanism is established. On the other hand, it effectively guides users in adjusting their gas demand, reducing load fluctuations, and enhancing system stability.

#### 1.2.2. Carbon Emissions Considerations in Natural Gas Dispatching

In the context of natural gas scheduling, in addition to considering the economic factors addressed in the above studies, it is essential to prioritize the system’s low-carbon benefits. This approach can better support the achievement of the “dual carbon” goals and play a critical role in promoting sustainable development. In IES, the management of carbon emissions within the natural gas subsystem is frequently addressed [19], with the ladder-type carbon trading mechanism (LTCTM) demonstrating significant potential in controlling these emissions [20]. To effectively constrain carbon emissions within the system, it is essential to develop differentiated management strategies tailored to users’ gas consumption scales and specific operating conditions. To the best of our knowledge, the LTCTM has not yet been incorporated into the natural gas downstream gas dispatching process for large-scale gas consumers.

Although natural gas is considered a low-carbon energy source, large-scale consumption still produces carbon emissions and environmental burdens. In the context of carbon emission control within NGS, existing studies mainly adopt a total control perspective. They rarely consider differentiated emission management across users of different scales and categories. This limitation reduces the adaptability of the models and makes it difficult to achieve low-carbon targets. This study proposes differentiated management schemes for different user types. Emission caps are imposed on residential users, while a LTCTM is introduced for non-residential users. These measures create more targeted and operational low-carbon scheduling strategies.

#### 1.2.3. Modeling User Behavior and Utility

In energy system scheduling research, user behavior modeling is a critical component for implementing demand-side management and load regulation. Existing studies often employ simplified utility functions to reduce model complexity. Although these methods provide a certain degree of theoretical guidance, their characterization of user behavior remains idealized. For example, Hassan et al. assumed that the utility function of users is non-decreasing and tends to stabilize after reaching a certain level. A quadratic equation was employed to quantify the utility of electricity consumption [21]. Su et al. determined the parameters based on expert opinions and employed a commonly used quadratic function to measure users’ utility for natural gas demand [22]. In practical applications, user utility is influenced by multiple factors such as price sensitivity and short-term behavior, exhibiting characteristics of non-uniform response and dynamic adaptation. Hence, developing a user utility model that realistically captures decision-making behavior and fully incorporates responses to price and load variations is essential to achieving more precise natural gas scheduling and more effective demand-side management.

Existing downstream scheduling models for natural gas employ simplified approaches in user behavior modeling. Utility functions are typically represented by static quadratic forms, which fail to capture user sensitivity to price variations and acceptance of load variations. As a result, the utility representation does not adequately reflect user preferences, reducing both the accuracy and practical value of optimization outcomes. An improved utility function is developed to optimize user participation decisions. It incorporates a price sensitivity coefficient and a load variation acceptance coefficient. An exponential formulation is applied to capture the nonlinear response of users to price and load variations. This enhances both the practical value and generalization ability of the utility function and overcomes the simplified assumptions in previous models.

In summary, this study integrates dynamic price signals and user behavior responses into an independent downstream natural gas scheduling framework. For different categories of consumers, the minimization of carbon emissions and emission costs is considered as environmental objectives. In addition, supplier costs and end-user welfare are included as indicators of economic performance. Based on these considerations, a demand-side low-carbon economic operation model for natural gas is established, tailored to heterogeneous user types. In addition, this study introduces an integrated EW-VIKOR approach to assist NSGA-III in multi-objective optimization. While NSGA-III generates a set of Pareto-optimal solutions, EW-VIKOR enables objective weighting of indicators and the selection of a unique compromise scheduling strategy. This methodological contribution ensures decision-makers are provided with a clear and actionable solution, thereby bridging the gap between theoretical optimization results and practical dispatch requirements.

### 1.3. Paper Organization

The structure of this paper is as follows. Section 2 introduces relevant theoretical methods in the natural gas sector, including the time-of-use elastic price response mechanism, ladder-type carbon trading, and user behavior modeling, with a particular focus on the objective functions and constraints of the scheduling model. Section 3 provides a detailed explanation of the solution methodology for the proposed model. Section 4 describes the data and parameters used in the simulation experiments and conducts a comprehensive analysis of the experimental results and associated strategies. Section 5 concludes the paper.

## 2. Theoretical and Modeling Approach to Low-Carbon Economic Operation of the Natural Gas Demand Side

### 2.1. Natural Gas Price-Based Demand Response Mechanism

The price-demand response mechanism refers to the use of price signals by the dispatch center to reduce demand or shift it to non-peak periods during gas consumption peak times. This mechanism effectively leverages the elastic portion of demand, optimizing natural gas consumption patterns, and reducing supply pressure during peak periods. In the energy sector, the traditional concept of price elasticity has been extended to the time dimension to describe user behavior under dynamic pricing conditions. This extension captures the temporal substitution effect, reflecting how a change in energy price at one point in time influences demand in subsequent periods. It provides a theoretical basis for natural gas dispatch strategy optimization by quantitatively describing the sensitivity of natural gas demand to price changes.

#### 2.1.1. Time-Based Price Elasticity of Natural Gas

The own price elasticity of natural gas measures the impact of price fluctuations on demand within the same time period. In the natural gas market, a price increase generally leads to a decrease in demand for the same time interval, making the own price elasticity coefficient typically negative. The calculation of the own price elasticity coefficient is given by Equation (1).

(1) $$\varepsilon_{ii}=\frac{∆g_{i}}{g_{i}^{0}}/\frac{∆p_{i}}{p_{i}^{0}}$$

Here, $\varepsilon_{ii}$ denotes the own price elasticity coefficient at time i, $∆g_{i}$ represents the change in load before and after the gas response at time i, and $g_{i}^{0}$ denotes the initial load at time i. $∆p_{i}$ refers to the change in natural gas price at time i, while $p_{i}^{0}$ represents the initial natural gas price for the time period. Under typical conditions, $\varepsilon_{ii}\leq 0$.

The cross-price elasticity of natural gas measures the impact of price changes at a given time point on the demand for natural gas at other time points. In the natural gas market, a price increase at one time point typically leads to a rise in demand during other periods, resulting in a cross-price elasticity value that is generally positive. The calculation of the cross-price elasticity coefficient is provided in Equation (2).

(2) $$\varepsilon_{ij}=\frac{∆g_{i}}{g_{i}^{0}}/\frac{∆p_{j}}{p_{j}^{0}}$$

Here, $\varepsilon_{ij}$ represents the cross-price elasticity coefficient. $∆p_{j}$ denotes the change in the natural gas price at time j. $p_{j}^{0}$ denotes the initial natural gas price for the time period. Typically, $\varepsilon_{ij}\geq 0$.

For flexible loads with reducible demand, own price elasticity reflects the user’s responsiveness to price increases. Reducible loads refer to the portion of demand that users are willing to curtail during periods of higher prices by reducing non-essential natural gas consumption. The cross-price elasticity is particularly significant in the context of flexible load, specifically within alternative loads. Alternative loads refer to the ability of users to meet the same demand by shifting consumption across different time periods.

#### 2.1.2. Dynamic Response Mechanism of Natural Gas Time-of-Use Price

For the hourly natural gas scheduling optimization, a full-period price elasticity matrix $E_{ij}$ is developed, consisting of own price elasticity and cross-price elasticity coefficients. This matrix provides a more comprehensive representation of how price variations influence user DR. Its mathematical formulation is presented in Equation (3).

(3) $$E_{ij}=\left[\begin{array}{cc} \begin{array}{cc} \varepsilon_{11} & \varepsilon_{12}\\ \varepsilon_{21} & \varepsilon_{22} \end{array} & \begin{array}{cc} \ldots & \varepsilon_{1j}\\ \ldots & \varepsilon_{2j} \end{array}\\ \begin{array}{cc} \ldots & \ldots \\ \varepsilon_{i1} & \varepsilon_{i2} \end{array} & \begin{array}{cc} \ldots & \ldots \\ \ldots & \varepsilon_{ij} \end{array} \end{array}\right]$$

In this study, natural gas DR is modeled using a time-of-use pricing mechanism based on peak, flat, and valley periods, aiming to capture the relationship between price variations and demand across different time intervals. By incorporating price elasticity theory, the response characteristics of natural gas demand are extended across multiple time periods. This approach establishes a three-dimensional elasticity coefficient matrix that encompasses both own price elasticity and cross-price elasticity. The matrix is used to analyze demand sensitivity to price changes within specific periods and its adjustment capability across time periods. The mathematical formulation is presented in Equation (4).

(4) $$E_{ts}=\left[\begin{array}{ccc} \varepsilon_{ff} & \varepsilon_{fp} & \varepsilon_{fg}\\ \varepsilon_{pf} & \varepsilon_{pp} & \varepsilon_{pg}\\ \varepsilon_{gf} & \varepsilon_{gp} & \varepsilon_{gg} \end{array}\right]$$

In this matrix, the diagonal elements $\varepsilon_{ff}$, $\varepsilon_{pp}$, $\varepsilon_{gg}$ represent the self-elasticity coefficients for the peak, flat, and valley periods, respectively. The off-diagonal elements $\varepsilon_{fp}$, $\varepsilon_{fg}$, $\varepsilon_{pg}$ correspond to the cross-elasticity coefficients between the peak, flat, and valley periods.

To map price variations to actual demand changes, Equation (5) provides the calculation of user demand in response to price signals.

(5) $$\left[\begin{array}{c} G_{f}\\ G_{p}\\ G_{g} \end{array}\right]=\left[\begin{array}{ccc} G_{f}^{0} & 0 & 0\\ 0 & G_{p}^{0} & 0\\ 0 & 0 & G_{g}^{0} \end{array}\right]\left[\begin{array}{ccc} \varepsilon_{ff} & \varepsilon_{fp} & \varepsilon_{fg}\\ \varepsilon_{pf} & \varepsilon_{pp} & \varepsilon_{pg}\\ \varepsilon_{gf} & \varepsilon_{gp} & \varepsilon_{gg} \end{array}\right]\left[\begin{array}{c} ∆p_{f}/p_{f}^{0}\\ ∆p_{p}/p_{p}^{0}\\ ∆p_{g}/p_{g}^{0} \end{array}\right]+\left[\begin{array}{c} G_{f}^{0}\\ G_{p}^{0}\\ G_{g}^{0} \end{array}\right]$$

In Equation (5), $G_{f}$, $G_{p}$, $G_{g}$ represent the natural gas loads after price adjustments during peak, flat, and valley periods. Similarly, $G_{f}^{0}$, $G_{p}^{0}$, $G_{g}^{0}$ denote the initial natural gas loads for the peak, flat, and valley periods.

By constructing an elasticity coefficient matrix and a time-of-use price DR model, the dynamic response characteristics of natural gas demand across different time periods can be analyzed more comprehensively.

### 2.2. Ladder-Type Carbon Trading Model

The Ladder-type carbon trading model (LTCTM) for natural gas is currently implemented primarily among non-residential users, particularly in the industrial and commercial sectors. In DSM, the introduction of the LTCTM serves as a crucial pricing approach to guide users in adjusting their loads. The implementation pathway is first reflected in the establishment of tiered thresholds. Using annual carbon dioxide emissions or comprehensive natural gas consumption as core indicators, different intervals are defined based on annual gas usage and average daily load levels. High-energy-consuming non-residential users are prioritized for inclusion in the regulatory scope. Building on this, carbon allowance prices increase incrementally through a laddered structure. When users exceed the baseline consumption range, they must purchase carbon emission rights at a higher cost, thereby creating a price-based deterrent. This mechanism works in coordination with demand response strategies. During peak load periods, laddered carbon costs and gas price signals combine to form a powerful incentive guiding user behavior, encouraging them to proactively reduce or shift natural gas consumption. Meanwhile, the ladder thresholds and pricing standards are determined by national and local carbon market regulations, and periodically adjusted according to industry characteristics, regional economic development, and variations in energy structures.

Participation in carbon trading corresponds to the difference between the actual carbon emissions from natural gas and the allocated carbon quota within a specific settlement period, as defined in Equation (6).

(6) $$∆Q=Q_{a}-Q_{quota}$$

Here, ∆Q represents the share of participation in carbon trading. $Q_{a}$ denotes the actual natural gas carbon emissions within a given period, while $Q_{quota}$ refers to the carbon emission quota for the same period.

In domestic energy markets, the free allocation method is commonly employed [23]. Drawing on this approach, we consider that the initial natural gas carbon quota is related to the natural gas consumption of non-residential users.

(7) $$Q_{quota}=\chi \sum_{t=1}^{T}G_{t}$$

Here, χ represents the carbon emission allocation coefficient for each unit of natural gas load. $G_{t}$ represents the natural gas load at time t, and *T* is the duration of a complete scheduling period.

The formula for calculating the actual carbon emissions of natural gas within a given settlement period is expressed in Equation (8).

(8) $$Q_{a}=\mu \sum_{t=1}^{T}G_{t}$$

Where μ represents the natural gas emission factor.

**Figure 1:**
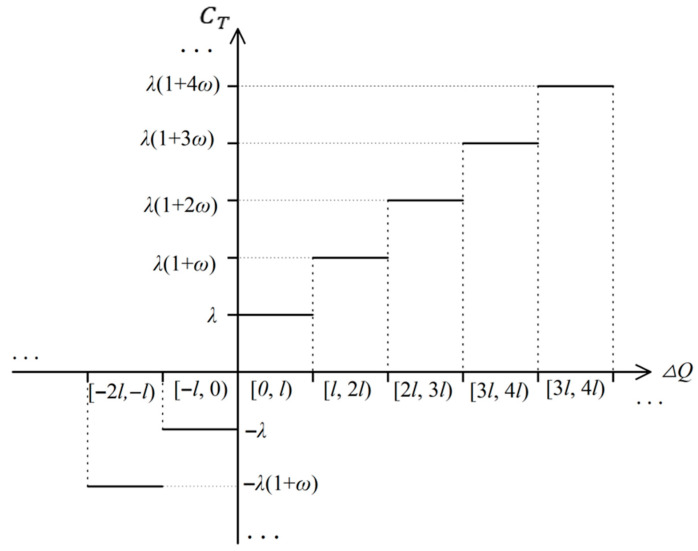
Ladder-type carbon trading mechanism diagram.

The LTCTM aims to optimize participants’ carbon emission behaviors in natural gas scheduling, balancing both economic and environmental objectives. Specifically, the mechanism divides total carbon emissions into multiple tiers based on emission levels, with each tier associated with a distinct carbon trading price. When emissions exceed the allocated quota, the resulting carbon trading cost, denoted as *C_T_*, is calculated using Equation (9). This formula is derived from the carbon pricing rules reported in Reference [24]. The specific LTCTM diagram is illustrated in Figure 1.

(9) $$C_{T}=\left\{\begin{array}{c} -\lambda \left(1+\omega \right)\left(-∆Q-l\right)-\lambda l,∆Q<-l\\ -\lambda \left(-∆Q\right),-l\leq ∆Q<0\\ \lambda \left(∆Q\right),0\leq ∆Q<l\\ \lambda \left(1+\omega \right)\left(∆Q-l\right)+\lambda l,l\leq ∆Q<2l\\ \lambda \left(1+2\omega \right)\left(∆Q-2l\right)+\left(2+\omega \right)\lambda l,2l\leq ∆Q<3l\\ \lambda \left(1+3\omega \right)\left(∆Q-3l\right)+\left(3+3\omega \right)\lambda l,3l\leq ∆Q<4l\\ \lambda \left(1+4\omega \right)\left(∆Q-4l\right)+\left(4+6\omega \right)\lambda l,4l\leq ∆Q \end{array}\right.$$

Where $C_{T}$ denotes the carbon trading cost, λ is the benchmark price for carbon trading. ω is the growth rate of the price. l represents the interval length.

### 2.3. User Behavior Modeling Methods

The ultimate goal of behavior modeling is to construct a utility function that accurately reflects the true preferences of users and integrate it into the scheduling model. In NGS, optimizing scheduling and pricing strategies allows users to receive stable gas supply services at lower prices, thereby enhancing their overall utility. The utility function is used to describe users’ demand preferences and consumption behavior patterns. The utility function is specified as a quadratic function of $G_{t}$. This form is chosen for its analytical tractability while providing a locally concave approximation consistent with the law of diminishing marginal utility within the relevant range of $G_{t}$ [21,22]. This implies that as gas consumption increases, the additional utility derived from each additional unit of gas decreases. Simultaneously, to ensure that the utility value remains non-negative, a constant offset term is introduced. This adjustment preserves the original utility order and prevents the occurrence of non-physical negative utility in low-consumption scenarios.

(10) $${U(G_{t})}_{base}=-a_{base}{G_{t}}^{2}+b_{base}G_{t}+c$$

Here, ${U(G_{t})}_{base}$ represents the general utility function, while $a_{base}$ and $b_{base}$ are the base benefit parameters, which take positive values and vary for different users. c is the offset constant.

The utility change in natural gas users not only reflects their sensitivity to price fluctuations but also captures the dynamic impact of load variations on their preferences. To more accurately capture the impact of price signals on user load behavior and its effect on utility, this study is inspired by multiple utility function modeling in reference [25]. Based on the existing utility function framework, a dynamic adjustment parameter in the form of an exponential function is introduced to address utility decision-making in the natural gas sector [26]. The calculation formula for the improved user utility function is presented in Equation (11).

(11) $$U(G_{t})=-a{G_{t}}^{2}+bG_{t}+c$$

The utility function parameters are redefined as functions of price variations and load responses (Equations (12) and (13)), incorporating coupled modeling of price sensitivity ($\lambda_{p}$) and load variation acceptance ($\lambda_{g}$). It is critical to note that the adoption of exponential functions for utility parameter refinement in natural gas consumer modeling demonstrates superior performance over conventional segmentation and S-functions in terms of behavioral adaptability and computational stability. The exponential function is simple in form, continuous, and differentiable, ensuring stable and efficient computation during the optimization process. Its parameters also possess strong economic interpretability, naturally capturing users’ behavioral responses, including asymmetry and diminishing marginal effects. To address these constraints, this study integrates nonlinear characteristics to adjust the sensitivity coefficients of price and load, allowing for a more accurate simulation of user response behavior in real-world scenarios.

(12) $$a=a_{base}\cdot exp(\lambda_{p}∆P_{t})\cdot \left(1+\lambda_{g}∆G_{t}\right)$$

(13) $$b=b_{base}\cdot exp({-\lambda }_{p}∆P_{t})\cdot \left(1+\lambda_{g}∆G_{t}\right)$$

Here, a and b are the adjusted benefit parameters, while $\lambda_{p}$ represents the price sensitivity coefficient, while $\lambda_{g}$ denotes the load variation acceptance coefficient. $∆P_{t}$ and $∆G_{t}$ represent the normalized values of price changes and load variations at a given time, respectively. By introducing normalization, values are converted into relative quantities, effectively eliminating the interference of absolute scale on the outcomes.

(14) $$∆P_{t}=\frac{\left|P_{t}^{after}-P_{t}^{0}\right|}{P_{t}^{0}}$$

(15) $$∆G_{t}=\frac{\left|G_{t}^{after}-G_{t}^{0}\right|}{G_{t}^{0}}$$

Here, $P_{t}^{after}$ and $G_{t}^{after}$ represent the price and load at time t after the scheduling optimization, respectively, while $P_{t}^{0}$ and $G_{t}^{0}$ denote the initial price and load at time t.

### 2.4. Cost Function of the Gas Supplier

The costs associated with natural gas scheduling primarily include pipeline gas procurement, gas storage and LNG acquisition costs, as well as the related pipeline distribution fees. Additionally, during peak load periods, the operation costs of compressors must be considered to maintain stable gas pressure. The cost function of the gas supplier must account for these factors and adjust dynamically based on market supply and demand, transportation, storage, and equipment scheduling.

(1) Procurement costs of pipeline gas, gas storage, and LNG.

The procurement costs of pipeline gas, gas storage, and LNG constitute the core components of the gas supplier’s cost structure. Pipeline gas is the primary source of supply, while gas storage plays a crucial role as a buffer resource to address demand fluctuations. LNG is used as a backup resource when the region is unable to supply gas in the short term. In addition to the base price of natural gas, gas storage involves operational costs associated with the injection, storage, and release processes, resulting in a slightly higher cost compared to pipeline gas. LNG, due to international market and transportation factors, incurs considerably higher short-term costs than the purchasing of both pipeline gas and gas storage.

(16) $$C_{pl}=\sum_{t=1}^{T}{P_{pl}G}_{pl,\;t}$$

(17) $$C_{sto}=\sum_{t=1}^{T}{P_{sto}G}_{sto,\;t}$$

(18) $$C_{lng}=\sum_{t=1}^{T}{P_{lng}G}_{lng,\;t}$$

Here, $C_{pl},\;C_{sto}$ and $C_{lng}$ represent the procurement costs of pipeline gas, gas storage, and LNG, respectively. $P_{pl}$, $P_{sto}$ and $P_{lng}$ denote the prices of pipeline gas, gas storage, and LNG. In the Chinese natural gas market, these prices exhibit the following relationship: $P_{pl}<P_{sto}<P_{lng}$. $G_{pl}$, $G_{sto}$ and $G_{lng}$ represent the quantities of gas procured from pipeline gas, gas storage, and LNG, respectively.

(2) Pipeline distribution costs

The transportation of natural gas is carried out through a pipeline system. The pipeline distribution costs are determined by factors such as the volume of gas transported, the transportation distance, and the utilization rate of the pipeline’s capacity.

(19) $$C_{ptp}=\sum_{t=1}^{T}{P_{ptp}G}_{t}L$$

Here, L represents the transportation distance, and $P_{ptp}$ denotes the transportation cost.

(3) Compressor operation costs

To ensure the stable delivery of natural gas to end users, compressors are activated when pipeline pressure is insufficient, in order to increase the gas’s kinetic energy and maintain stable pressure. For the purpose of simplifying the operational cost of compressors, only the energy consumption cost is considered. Other indirect costs, such as maintenance, depreciation, and labor costs, are beyond the scope of this study. The specific calculation is given by Equation (20) [27].

(20) $$C_{comp}=\sum_{n=1}^{n_{c}}\left(W_{p,n}{t_{period}p}_{ce}\right)$$

Here, $C_{comp}$ represents the compressor operation cost. $W_{p,n}$ denotes the power of the n-th compressor, while $t_{period}$ refers to the operational duration of the n-th compressor within a given scheduling period. $p_{ce}$ represents the energy cost coefficient of the compressor, and $n_{c}$ indicates the number of compressors in operation. The calculation of compressor power is provided in Section S1 of the Supplementary Materials.

### 2.5. Load Volatility Function

Significant fluctuations in natural gas demand can reduce the stability and security of the entire scheduling system. Therefore, it is essential to account for load fluctuations in the scheduling optimization process to quantify the associated instability costs of the system [17]. Natural gas load volatility refers to the amplitude and frequency of load variations within a scheduling period. The load volatility function is used to quantitatively assess the dynamic characteristics of demand over time, capturing the variation behaviors of peak, steady, and off-peak loads.

(21) $$V_{load}=\frac{\sqrt{\frac{1}{T}\sum_{t=1}^{T}{\left(G_{t}-\bar{G}\right)}^{2}}}{\frac{1}{T}\sum_{t=1}^{T}G_{t}}$$

Here, $V_{load}$ represents the load volatility function, and $\bar{G}$ denotes the average natural gas load over a scheduling period.

### 2.6. Model Construction

**Figure 2:**
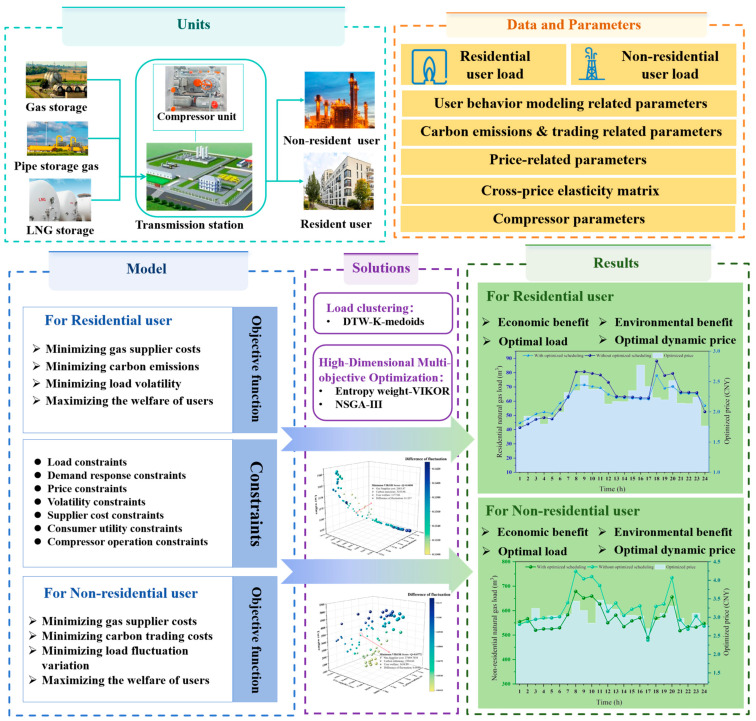
The overall schematic for low-carbon economic dispatch of terminal natural gas targeting residential and non-residential users.

This study focuses on urban natural gas end users and investigates the optimization of point-to-point low-carbon gas supply under a flexible demand-side response mechanism. By integrating dynamic price incentives with user behavior analysis, it aims to balance the economic and environmental objectives of residential and non-residential user groups. The structural framework of the natural gas low-carbon economic scheduling (NGLCES) model is illustrated in Figure 2. In terms of gas source dispatch, the gas supply at the distribution station follows a multi-level priority strategy: first, the pipe storage gas is utilized; second, the stored gas in gas storage is dispatched; and finally, as an emergency backup measure, LNG procurement is considered to maintain gas supply stability in the system. The framework diagram consists of four main modules: units, data and parameters, model, solutions, and results. The system unit shows the physical system of the research object, including natural gas supply sources, stations, and residential and non-residential users. The data and parameters module provides the required inputs for the model, such as user load data, behavioral parameters, price and carbon emission parameters, and the cross-price elasticity matrix. The modeling module formulates the objective functions and constraints for different types of users. By applying load clustering and multi-objective optimization, the model generates user response and system scheduling results. The results module presents the optimized load curves, dynamic pricing, and the associated economic and environmental benefits. Overall, the diagram illustrates the complete procedure from data input to optimization and result output.

#### 2.6.1. Objective Function

This study focuses on residential and non-residential natural gas users and develops a NGLCES model that integrates dynamic pricing signals and an improved user utility function, using gas price as the decision variable. It is important to note the following aspects: (1) The welfare of natural gas users refers to the total benefit that users derive from consuming natural gas, typically defined as the difference between user utility and user expenditure [28]. The ultimate goal for users is to maximize their welfare, which involves optimizing the difference between utility and expenditure by adjusting natural gas consumption and consumption patterns under specific price and constraint conditions. (2) In the modeling of carbon emission management, the study focuses on setting differentiated emission constraints for different user types based on natural gas consumption patterns. For residential users, natural gas demand is limited, and their contribution to total carbon emissions is relatively small. Therefore, the calculation of emissions only considers the relation between gas consumption and the carbon emission factor, and the objective function includes emission minimization without placing these users in the context of carbon market participation. In contrast, non-residential users have higher energy consumption and thus bear more significant emission responsibilities. Therefore, this paper incorporates a laddered carbon emissions trading cost mechanism based on benchmark quotas into the model to reflect the environmental costs incurred at different energy consumption levels. The design of the mechanism draws on existing policies in the power sector, but its function lies mainly in representing environmental objectives within the multi-objective optimization model rather than in directly mapping real carbon market regulations.

In summary, for residential users, the objective function primarily considers the minimization of supplier costs, carbon emissions, load volatility, and the maximization of user welfare, as shown in Equation (22).

(22) $$\left\{\begin{array}{l} min\;\left[C_{pl}+C_{sto}+C_{lng}+C_{ptp}\right]\\ min\;\mu \sum_{t=1}^{T}G_{t}\\ min\;V_{load}\\ max\;\left[U(G_{t})-\sum_{t=1}^{T}{P_{t}G}_{t}\right] \end{array}\right.$$

For non-residential users, the objective function focuses on the minimization of supplier costs, ladder-type carbon trading costs, load volatility, and the maximization of user welfare, as shown in Equation (23).

(23) $$\left\{\begin{array}{l} min\;\left[C_{pl}+C_{sto}+C_{lng}+C_{ptp}\right]\\ min\;C_{T}\\ min\;V_{load}\\ max\;\left[U(G_{t})-\sum_{t=1}^{T}{P_{t}G}_{t}\right] \end{array}\right.$$

#### 2.6.2. Constraints

(1) Natural gas load constraints

The core principle of load management is to ensure that the natural gas supply from pipeline transportation, gas storage facilities, and LNG sources can sufficiently meet real-time natural gas demand, as expressed in Equation (24). The coordinated operation of natural gas from various supply sources is essential to mitigate risks at the supply terminals.

(24) $$G_{pl,t}{+G}_{sto,t}+G_{lng,t}=G_{d,t}$$

Here, $G_{d,t}$ represents the natural gas demand of the user at time t.

(2) Natural gas demand response constraints

Over a complete scheduling period, the total natural gas load after DR typically does not exceed the load prior to the response. Additionally, the magnitude of load variation must remain within the allowable maximum load change limits. The specific constraints are expressed in Equation (25).

(25) $$\left\{\begin{array}{c} G^{min}\leq \sum_{t=1}^{T}G_{t}\leq G^{max}\\ G^{max}=\sum_{t=1}^{T}G_{t}^{0}\;\;\;\;\;\;\;\;\;\;\;\;\\ G^{min}=0.85\sum_{t=1}^{T}G_{t}^{0}\;\;\; \end{array}\right.$$

In this context, $G_{t}^{0}$ represents the initial load of natural gas at time t, while $G^{0}$ denotes the mean value of the initial gas load.

(3) Natural gas price constraints

Under the dual influence of market demand and government regulation, an upper limit must be set on natural gas prices. Additionally, time-of-use pricing during peak, flat, and valley periods should strictly adhere to the established formal models, as defined in Equation (26).

(26) $$\left\{\begin{array}{c} 0\leq P_{g}\leq P_{p}\leq P_{f}\\ P_{g}\leq P_{0}\leq P_{f}\leq P_{up} \end{array}\right.$$

Here, $P_{g},\;P_{p}$ and $P_{f}$ represent the prices during valley, flat, and peak periods, respectively, while $P_{0}$ denotes the initial gas price. In China, natural gas prices are subject to a regulatory upper limit $P_{up}$, determined by policy requirements and market conditions.

(4) Natural gas volatility constraints

To maintain the stability and efficiency of the NGS, the volatility after optimization and scheduling should be lower than the levels before optimization.

(27) $$\sum_{t=1}^{T}V_{load}^{0}\geq \sum_{t=1}^{T}V_{load}$$

Here, $V_{load}^{0}$ represents the load volatility prior to the DR.

(5) Natural gas supplier cost constraints

To align with the cost objectives of the gas supplier, the cost following the optimized scheduling should be lower than that incurred prior to the optimization.

(28) $$\sum_{t=1}^{T}C_{cost}^{0}\geq \sum_{t=1}^{T}C_{cost}$$

Here, $C_{cost}^{0}$ represents the cost incurred by the gas supplier prior to the DR.

(6) Natural gas consumer utility constraints

For natural gas end-users, the utility value after optimized scheduling should be higher than before.

(29) $$\sum_{t=1}^{T}U(G_{t})\geq \sum_{t=1}^{T}U(G_{t}^{0})$$

(7) Compressor operation constraints

The operation of the compressor is contingent on the pressure within the pipeline. When pressure is sufficient, the natural gas flows directly through the bypass valve, and the compressor remains inactive. However, when the pipeline pressure is insufficient, natural gas is directed to the station’s compressor for compression [29]. In this case, it is essential to ensure that the gas flow rate remains constant and matches the load for the given scheduling period. The constraint is expressed in Equation (30).

(30) $$\left\{\begin{array}{c} G_{d,t}=S_{bp,t}G_{bp,t}+S_{com,t}G_{com,t}\\ G_{com,t}=\sum_{n=1}^{nc}G_{com,cn,t}\\ S_{bp,t}+S_{com,t}=1 \end{array}\right.$$

Here, $S_{bp,t}$ and $S_{com,t}$ represent the opening status indicators for the bypass valve and compressor valve at time t, where a value of 0 denotes the closed state, and a value of 1 denotes the operational state. $G_{bp,t}$ and $G_{com,t}$ represent the natural gas flow rates through the bypass valve and compressor valve, respectively, at time t.

#### 2.6.3. Complete Optimization Model

To provide a clearer representation of the proposed framework, the optimization objectives and constraints from Section 2.6.1 and Section 2.6.2 are integrated into a unified multi-objective optimization model, as expressed in Equation (31). The definitions of the model variables are presented in the nomenclature table in Section S2 of the Supplementary Materials.

(31) $$\left\{\begin{array}{l} min\;\{f_{1},f_{2},f_{3},f_{4}\}\\ f_{1}=C_{pl}+C_{sto}+C_{lng}+C_{ptp}\\ f_{2}=\left\{\begin{array}{c} \begin{array}{cc} \mu \sum_{t=1}^{T}G_{t} & for\;residential\;users \end{array}\\ \begin{array}{cc} C_{T} & for\;nonresidential\;users \end{array} \end{array}\right.\\ {f_{3}=V}_{load}\\ f_{4}=-\left[U(G_{t})-\sum_{t=1}^{T}{P_{t}G}_{t}\right]\\ s.t.\\ G_{pl,t}{+G}_{sto,t}+G_{lng,t}=G_{d,t}\\ G^{min}\leq \sum_{t=1}^{T}G_{t}\leq G^{max}\\ 0\leq P_{g}\leq P_{p}\leq P_{f}\\ P_{g}\leq P_{0}\leq P_{f}\leq P_{up}\\ \sum_{t=1}^{T}V_{load}^{0}\geq \sum_{t=1}^{T}V_{load}\\ \sum_{t=1}^{T}C_{cost}^{0}\geq \sum_{t=1}^{T}C_{cost}\\ \sum_{t=1}^{T}U(G_{t})\geq \sum_{t=1}^{T}U(G_{t}^{0})\\ G_{d,t}=S_{bp,t}G_{bp,t}+S_{com,t}G_{com,t}\\ S_{bp,t}+S_{com,t}=1 \end{array}\right.$$

To address the limited applicability of natural gas scheduling models across different regions and consumer types, the proposed model is designed with a strong focus on generalization and transferability during its development stage. The model inputs include multiple parameterized variables, such as user load profiles, price elasticity coefficients for different user categories, and natural gas prices. When the model is applied to different scenarios, it achieves adaptability by adjusting parameter values without altering the core framework or solution algorithm. For load pattern recognition, the model applies a clustering method (see Section 3.1 for details) to classify peak, valley, and flat periods. This approach captures the temporal characteristics and variations in user gas consumption. It effectively identifies diverse load patterns caused by regional differences, seasonal changes, and user scale. Based on clustering results, the model adaptively generates time-of-use pricing schemes that balance economic and environmental objectives. This approach guides users to optimize gas consumption patterns, achieving coordinated improvements in peak shaving and valley filling while enhancing overall system efficiency. Through this parameter-driven and data-adaptive mechanism, the model does not rely on data from a single region and can maintain strong generalization and applicability across diverse regions and user groups.

## 3. Model Solving Method

### 3.1. Data-Driven Method to Identify Peak, Flat and Valley Periods

**Figure 3:**
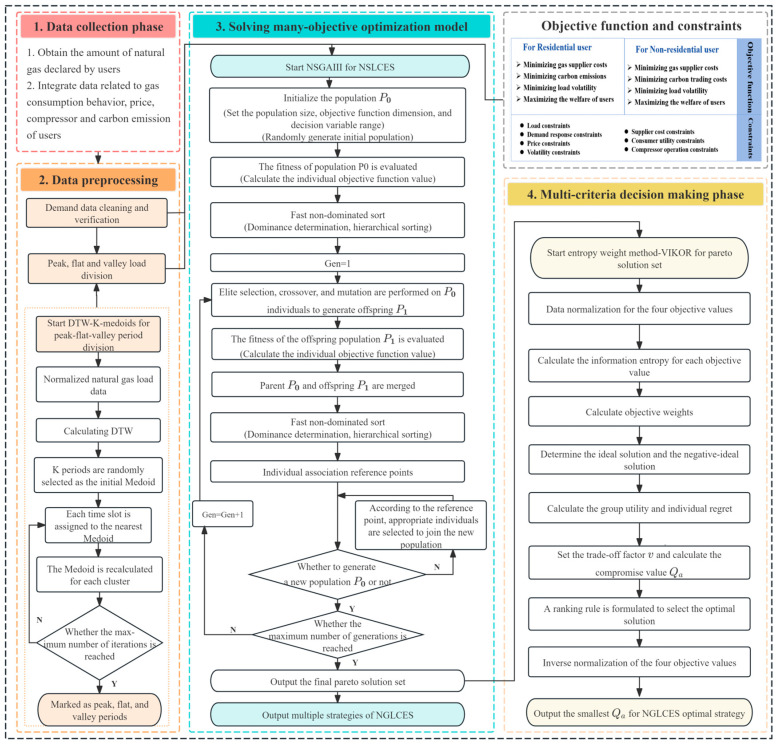
Flowchart of the model solution method.

There are significant differences in natural gas demand among users across different time periods. This study employs peak, flat, and off-peak time segmentation along with a time-of-use price elasticity matrix to capture the price sensitivity and demand response characteristics of different periods, thereby enabling scheduling strategies to more accurately align with actual demand variations. Existing time-of-use pricing strategies are predominantly based on empirically determined threshold-based manual segmentation methods, which overlook the structural differences in gas consumption patterns among different user types, including the temporal distribution of usage behavior, load fluctuations, and price elasticity. For instance, residential gas consumption peaks are concentrated in the morning and evening due to daily living routines, whereas non-residential users exhibit intermittent peaks influenced by production schedules. Therefore, this study employs the dynamic time warping k-medoids (DTW-K-medoids) clustering method to conduct temporal similarity analysis and clustering of load data [30]. The detailed process flowchart is shown on the left side of Figure 3. The similarity between time series data is measured using the DTW algorithm (Equation (32)), which is well-suited for pattern recognition in time segmentation.

(32) $${DTW}_{t_{1},t_{2}}=|G_{{it}_{1}}-G_{{jt}_{2}}|+min\{{DTW}_{t_{1}-1,t_{2}},{DTW}_{t_{1},t_{2}-1},{DTW}_{t_{1}-1,t_{2}-1}\}$$

$G_{{it}_{1}}$ represents the natural gas load at time $t_{1}$ on the i-th day, while $G_{{jt}_{2}}$ denotes the natural gas load at time $t_{2}$ on the j-th day.

### 3.2. Multi-Objective Optimization Method Integrating Multi-Criteria Decision-Making Algorithms

In high-dimensional multi-objective optimization problems (MOPs), solving and characterizing the Pareto optimal solution set is one of the core research tasks. This study constructs a high-dimensional multi-objective optimization model for natural gas scheduling, applicable to both residential and non-residential users. In practical scheduling processes, multiple objective functions are difficult to optimize simultaneously and exhibit a certain degree of conflict. In addition, the computational complexity of high-dimensional optimization problems is high, and the traditional optimization method of transforming multi-objectives into a single objective is difficult to solve effectively. To address the aforementioned issues, this study integrates multi-criteria decision-making (MCDM) algorithms into high-dimensional multi-objective optimization methods to achieve a rational trade-off among different objectives while enhancing the efficiency and accuracy of the optimization process.

This study employs the non-dominated sorting genetic algorithm III (NSGA-III), which utilizes a non-dominated sorting mechanism and a reference point-guided approach to identify a set of mutually non-dominated solutions within the solution space, thereby constructing the Pareto front [31]. Specifically, during the solution process, it is first necessary to establish a precise mathematical formulation and definition for each objective function. Additionally, the feasible solution space must be constrained through appropriate constraint conditions to ensure solution validity. This process ensures that the obtained solution set has no significant potential for further improvement across all objectives.

Although NSGA-III provides clear advantages, its computational complexity may still increase significantly when the number of objectives grows, or the problem size expands. To address this issue, the model adopts two strategies. First, it applies precise modeling of each objective function and introduces constraints to effectively limit the feasible solution space, which reduces the computational burden. Second, it integrates a MCDM approach based on the entropy weight (EW) method and VIKOR [32], which allows the selection of a unique compromise solution from the Pareto front. This not only alleviates the computational effort in practical scheduling decisions but also strengthens the applicability of NSGA-III in large-scale and high-dimensional systems. VIKOR demonstrates superior capability in reconciling conflicts among multiple objectives and ensuring reasonable weight allocation, compared to traditional MCDM methods such as TOPSIS or AHP [33]. The steps of the EW-VIKOR method are provided in Section S3 of the Supplementary Materials. The VIKOR presented in this paper introduces a decision-making mechanism factor v to balance the preferences between the overall optimal solution and the individual optimal solutions. The load distribution scheme corresponding to the smallest value of $Q_{a}$ is selected as the final scheduling solution. Figure 3 presents the complete solution process of the model, including data input, data preprocessing, optimization solving, and result output. The model integrates multi-objective optimization with a decision-making mechanism to generate optimal scheduling strategies under different demand response scenarios.

## 4. Experiment and Analysis

### 4.1. Data Description and Parameter Description

**Figure 4:**
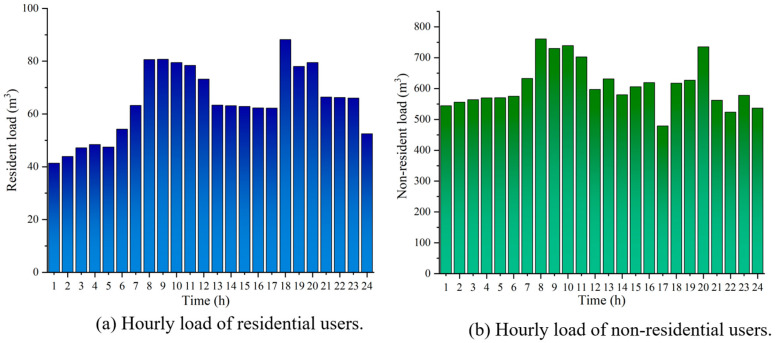
Hourly natural gas load of residential and non-residential users.

To validate the proposed scheduling model, this study utilizes branch natural gas load data from a gas station in Xi’an, Shaanxi Province in 2020. The dataset captures the load variations in residential and non-residential users over 24 h. To meet data confidentiality requirements, the user data employed consists of field station data that has undergone linear scaling processing. The scaling only adjusts the absolute load range without altering structural features of the time series, such as peak-valley distribution and fluctuation patterns. As the model emphasizes relative load variations and demand response behavior, the use of scaled data does not affect the robustness of the results or the reliability of the conclusions. This study assumes that compressors need to be activated during peak gas demand periods to pressurize the pipeline gas. The detailed compressor model and parameters are provided in Table S1 of Supplementary Materials. Moreover, natural gas suppliers formulate supply plans at least one scheduling period in advance to satisfy the projected gas demand of regional users. The specific load data used is illustrated in Figure 4.

Residential load refers to the natural gas consumption required by households within the supply area. Residential demand is usually small in scale and is supplied indirectly through the urban distribution network. Non-residential users typically refer to large natural gas-consuming enterprises that are major emission sources or high-energy-consumption entities. Their natural gas supply needs are usually met through direct supply contracts or medium-to-high-pressure direct supply methods. The scheduling model designed in this study has a time unit of 1 h and a cycle of 24 h. The carbon emission and trading model, as well as the user behavior model parameters for scheduling natural gas load for residential and non-residential users, are provided in Table 1 [34,35,36,37]. To enhance the empirical validity of utility function modeling, $\lambda_{p}$ and $\lambda_{g}$ are determined with reference to relevant literature [25], and further calibrated based on the observed variations in regional natural gas consumption. It is worth noting that the adjustment of gas consumption behavior by end users exhibits a certain degree of rigidity, as their flexibility is constrained by various factors such as daily routines and industrial processes. Here, $\lambda_{g}$ represents the user’s load adjustment acceptance degree, influenced by infrastructure and gas consumption patterns. Based on real-world conditions and user willingness constraints, the upper limit of load variation acceptability is set to no more than 0.1 to reflect the maximum adjustable capacity in actual gas consumption. Meanwhile, $\lambda_{p}$ represents the intensity of users’ response to price variations, with its upper limit set at 0.1. Excessively high $\lambda_{p}$ causes load inversion, failing to achieve peak shaving and valley filling while inducing user response distortion. $\lambda_{p}$ and $\lambda_{g}$ serve as relative behavioral modeling parameters designed to reflect the comparative differences between distinct user types, rather than aiming for precise quantitative results. Their ranges may vary across regions or user groups, depending on infrastructure conditions, gas consumption patterns, and users’ willingness to adjust demand. Due to the lack of complete household-level consumption monitoring data and user behavior surveys, this study assumes, based on historical natural gas consumption patterns in Xi’an, that the values of $\lambda_{p}$ and $\lambda_{g}$ for residential users are 0.05 and 0.02, while for non-residential users they are 0.08 and 0.04. Residential users follow relatively fixed daily routines, and their gas demand is characterized by low substitutability and limited reducibility. As a result, their flexibility in load adjustment remains constrained. In contrast, non-residential users are more sensitive to price changes, primarily due to the higher adaptability of their production schedules. Therefore, the coefficient of price sensitivity and load variation acceptance of residential users is usually lower than that of non-residential users, which also reflects the difference in demand response characteristics of different user categories. Price-related information is presented in Table 2, with data sourced from the natural gas pricing reform plan released by the Xi’an Development and Reform Commission, as well as the tracking and rating report from Xi’an Infrastructure Investment Group.

**Figure 5:**
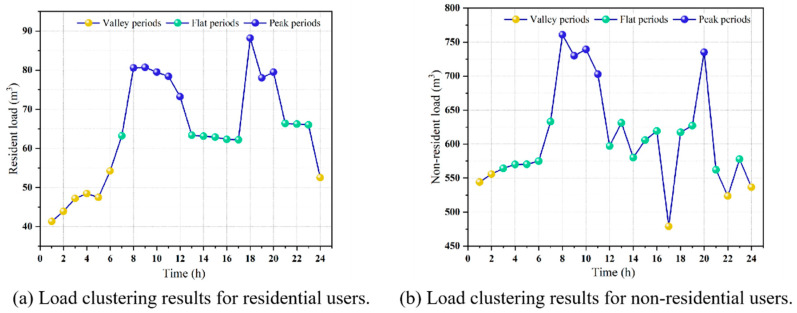
Time-of-day clustering results based on DTW-K-medoids.

The time-division results of peak, flat, and valley obtained by using DTW-K-medoids in this study are shown in Figure 5. For residential users, the peak periods are defined as 8:00–12:00 and 18:00–20:00, the flat periods as 7:00, 13:00–17:00, and 21:00–23:00, and the valley periods as 1:00–6:00 and 24:00. For non-residential users, the peak periods are 8:00–11:00 and 20:00, the flat periods are 3:00–7:00, 12:00–16:00, 18:00–19:00, 21:00, and 23:00, while the valley periods are 1:00–2:00, 17:00, 22:00, and 24:00. Through a data-driven approach, it was identified that residential users exhibit a “bimodal” pattern (8:00–12:00, 18:00–20:00), while non-residential users display a “pulsed” peak period (8:00–11:00, 20:00). This segmentation provides a temporal benchmark for the subsequent implementation of differentiated scheduling strategies, enhancing the alignment between dynamic price signals and the actual energy usage patterns of users. Regarding the price elasticity coefficients for different time periods, the literature calculates the own price elasticity coefficient for the residential sector in China to be −0.233 [38]. The findings align with the consumption characteristics of residential gas users in China. The price elasticity of demand for the non-residential sector in China, as presented in this literature, exhibits behavior contrary to the conventional law of demand due to the limitations imposed by insufficient energy supply. However, the energy resources in Shaanxi Province, selected for this study, are relatively abundant. It is home to one of China’s key natural gas production regions and receives external supplies through the national natural gas backbone network. Consequently, the price elasticity of the non-residential sector aligns with the conventional demand law. Due to the lack of specific statistical data and research on the price elasticity coefficients for non-residential users in Shaanxi Province, this study estimates the required price elasticity coefficients based on the findings from the literature [39,40]. The elasticity coefficients for the time-of-use pricing scheme adopted in this study are presented in Table 3.

### 4.2. Experimental Results and Strategies

**Figure 6:**
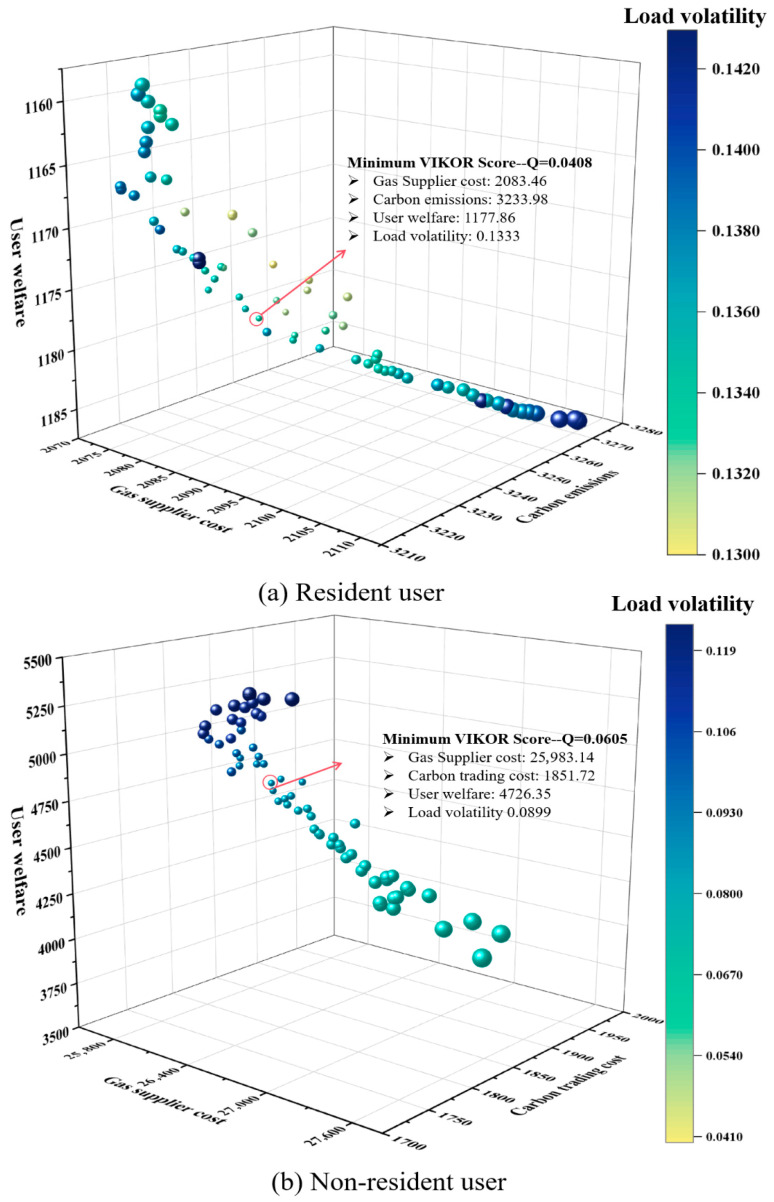
Pareto optimal solution set of the NGLCES model.

Based on the proposed NGLES model, the dispatch center applies the high-dimensional multi-objective optimization method, as described in Section 3, to generate a Pareto-optimal solution set. This set represents trade-off solutions under four objectives: cost minimization, carbon emission and trading minimization, user welfare maximization, and load fluctuation minimization, reflecting Pareto efficiency. Subsequently, the dispatch center selects a unique optimal scheduling strategy from the solution set using a MCDM approach. With a decision-making mechanism factor of 0.5, this strategy balances collective utility and individual regret, achieving a synergistic compromise between economic environmental benefits and system stability. The entire process strictly adheres to the principle of uniqueness in single-dispatch strategies, ensuring the global optimality of dispatch instructions and the consistency of execution. For residential users, the scheduling model considers minimizing gas supplier costs, carbon emissions, and load volatility while maximizing residential user welfare. The trade-off solution is indicated by the red markers in Figure 6a. For non-residential users, the scheduling model seeks to minimize gas supplier costs, ladder-type carbon trading costs, and load volatility, while maximizing user welfare. Figure 6b highlights the resulting trade-off solution with a red marker.

**Figure 7:**
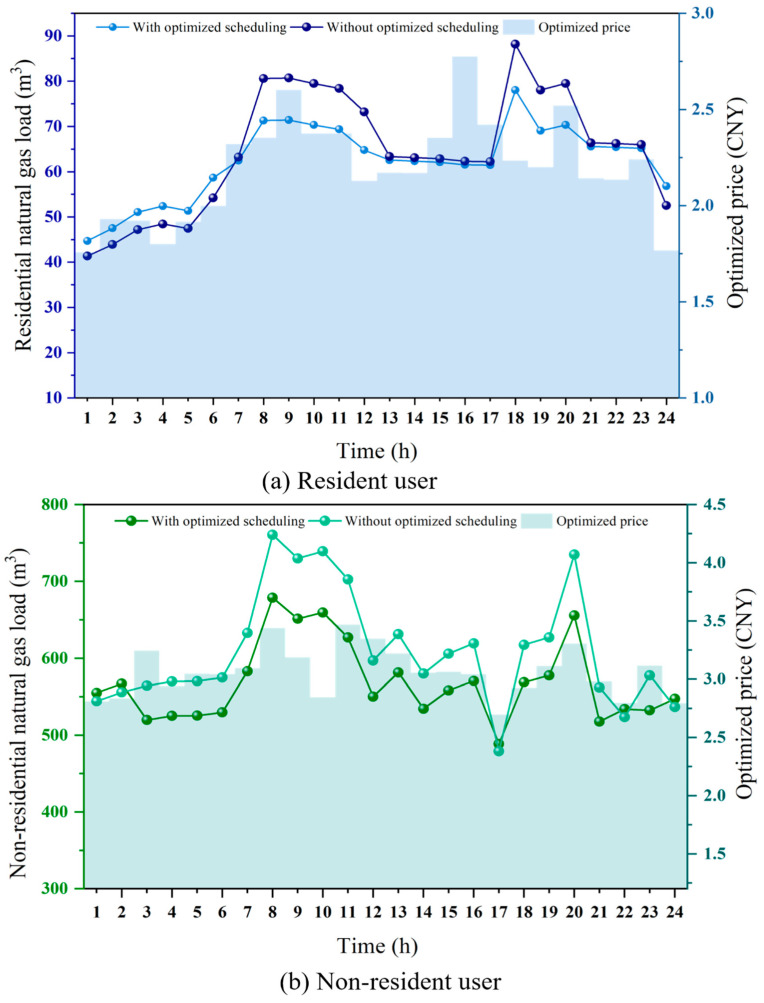
The load and dynamic pricing after scheduling optimization.

In order to evaluate the practical scheduling application of the NGLES model for both residential and non-residential users, the NSGA-III-EW-VIKOR is employed as the solution model, with a decision-making mechanism factor of 0.5 used to determine the unique optimal scheduling scheme. Due to the significant differences in gas consumption behavior between residential and non-residential users, the model incorporates differentiated parameter settings for the user utility functions during the user behavior modeling process, aiming to more accurately reflect the decision-making characteristics of different user types. The price sensitivity and load variation acceptance of residential users are set at 0.05 and 0.02, respectively, based on the actual response characteristics of residential users to fluctuations in natural gas prices and load variations. In contrast, non-residential users exhibit a more significant response to price and load changes compared to residential users. Therefore, their price sensitivity and load variation acceptance are set at 0.08 and 0.04, respectively, to better capture their gas consumption elasticity. Figure 7a and Figure 7b illustrate the dynamic natural gas prices and the hourly gas consumption variations for residential and non-residential users under the optimized scheduling scenario, respectively.

Regarding natural gas pricing, before optimization, the terminal price for residential natural gas was 2.18 CNY, while that for non-residential users was 3.04 CNY. After optimization, the real-time gas price exhibited significant temporal differentiation, effectively reflecting the guiding role of price signals in influencing user gas consumption behavior. As shown in Figure 7a, during the peak gas consumption periods of residential users (08:00–12:00, 18:00–20:00), the dynamic pricing increases from the fixed price of 2.18 CNY to a maximum of 2.78 CNY at 16:00. Considering that dynamic price signals can effectively regulate peak gas consumption among residential users, the total natural gas load is reduced from 1548.96 m^3^ to 1495.69 m^3^, representing a reduction of 53.27 m^3^. This alleviates the pipeline network load pressure and the gas allocation burden during the scheduling process. Meanwhile, the load volatility decreased from 0.2059 to 0.1333, indicating that the proposed NGLCES model significantly enhances the stability of natural gas supply. The peak gas consumption periods for non-residential users occur between 8:00–11:00 and at 20:00. As shown in Figure 7b, the dynamic pricing mechanism increases the fixed price of 3.04 CNY to a maximum of 3.47 CNY at 11:00. The total natural gas load decreased from 14,638.10 m^3^ to 13,640.59 m^3^, representing a reduction of 997.51 m^3^. Additionally, a portion of the flexible load was shifted from peak periods to valley periods, thereby reducing the peak-to-valley difference. Meanwhile, the natural gas volatility for non-residential users decreased from 0.1221 to 0.0899. This result indicates that appropriate price regulation can effectively mitigate load fluctuations and enhance the operational stability of the system. Non-residential users contribute more to both absolute and relative load reductions and serve as the main source of lowering system peak demand and total consumption. Residential users show smaller reductions in absolute terms, but they play a more significant role in smoothing hourly fluctuations. This supports scheduling stability and reduces short-term balancing costs. The experimental results indicate that the optimized dynamic pricing mechanism achieves dynamic regulation of supply and demand balance between residential and non-residential users, providing a crucial theoretical foundation for the further development of real-time pricing policies in the natural gas sector. The findings validate the effectiveness of dynamic price signals and user behavior modeling in multi-objective optimization scheduling.

**Figure 8:**
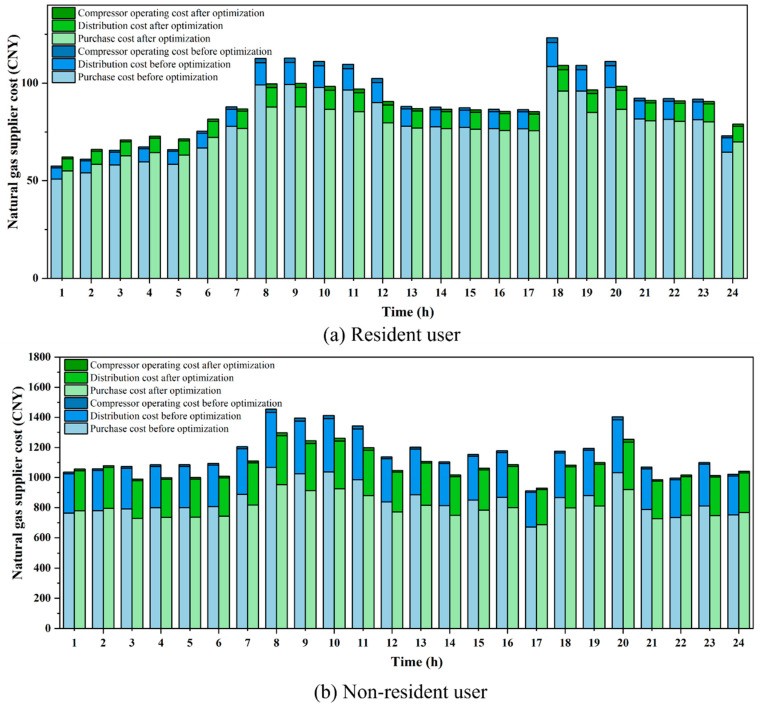
The gas supplier’s purchasing cost, distribution cost, and compressor operating cost before and after scheduling optimization.

In terms of economic benefits, the gas supplier costs and user welfare after scheduling optimization are calculated based on price adjustments and load changes. The gas supplier purchases gas in advance based on the users’ consumption plans. Table 4 presents the gas supply costs, revenues, and profits of the gas supplier required to meet the demand of residential users. The proposed scheduling optimization yields a cost saving of 74.64 CNY for the gas supplier. This reduction includes a decrease of 65.52 CNY in gas procurement costs, 7.45 CNY in distribution costs, and 1.67 CNY in compressor operation costs. The corresponding hourly gas purchase costs, distribution costs, and compressor operation costs before and after scheduling optimization are illustrated in Figure 8a. Additionally, this study calculates the revenue and profit of the gas supplier, with the optimized results showing an increase of 11.49 CNY in profit compared to the pre-optimization scenario. After optimizing the scheduling strategy, the gas supply cost for suppliers serving residential users increased during valley periods. However, during flat and peak periods, the cost after scheduling optimization significantly decreased, with the most notable reduction observed during the peak periods. This is attributable to the price signals encouraging users to shift part of their gas consumption to the lower-priced off-peak periods, thereby reducing the demand during peak periods. This optimization not only improved gas usage behavior but also effectively alleviated the supply pressure during peak periods, reducing the gas procurement costs and distribution losses during these times.

Table 5 presents detailed information regarding the gas supplier costs, revenue, and profit for non-residential users. After the optimization of the scheduling strategy, the total gas supply cost incurred by the gas supplier for non-residential user gas scheduling was reduced by 1901.39 CNY. Specifically, the costs for gas procurement, transportation and distribution, and compressor operation were reduced by 1399.5 CNY, 478.81 CNY, and 23.08 CNY, respectively. The gas supplier’s revenue decreased by 746.32 CNY compared to the pre-optimization scenario. The corresponding comparison of hourly gas purchase costs, gas distribution costs, and compressor operating costs before and after the scheduling optimization is illustrated in Figure 8b. For non-residential users, particularly large industrial users, there is a distinct difference in gas demand compared to residential users. These users exhibit a higher sensitivity to price signals and have relatively flexible gas consumption schedules. Therefore, after the optimization of the scheduling, the gas supply costs for the supplier significantly decrease during flat and peak periods, while costs during the valley period experience a slight increase. This phenomenon is primarily attributed to the lower natural gas prices during valley periods, which allow large industrial users to flexibly adjust their operating hours or production processes, thereby shifting some of their energy consumption to lower-price periods. This load-shifting strategy not only helps users reduce gas consumption costs but also effectively alleviates the supply pressure during peak periods. It is noteworthy that the gas supplier’s revenue decreased after optimization. This is attributed to non-residential users adjusting their gas consumption schedules to periods with lower gas prices or switching to alternative energy sources, resulting in reduced purchasing revenue for the gas supplier, thereby leading to a decline in the supplier’s profit. In response to this situation, policy incentives are required to compensate gas suppliers in order to offset the economic losses incurred from ensuring the stability of the gas scheduling system and the safety of transportation. In addition, the operating costs of compressors during peak periods are higher compared to off-peak and valley periods. This is primarily due to the significant increase in gas demand during peak periods, which necessitates the use of compressors to maintain the stability of the gas pipeline network, thereby ensuring the normal gas supply during peak times.

**Figure 9:**
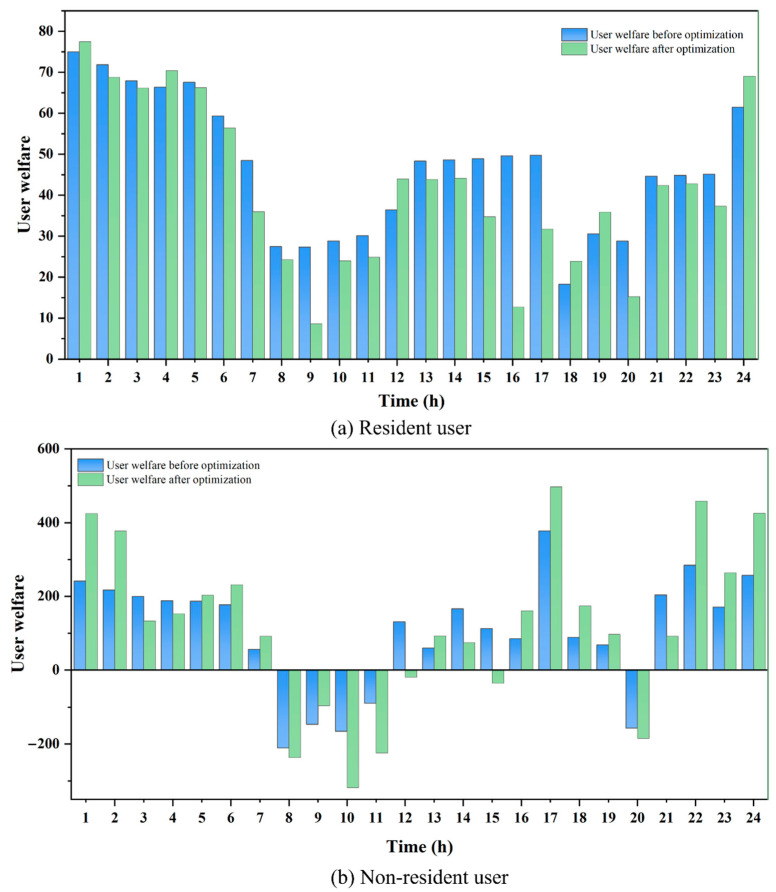
User welfare before and after scheduling optimization.

The gas purchase costs, utility, and welfare for residential users are presented in Table 6. Although the optimized user utility is 10.87 lower than before, the amount spent on gas purchase has decreased by 63.15 CNY. According to Equation (24), the total user welfare is 52.28 units higher than the welfare prior to optimization. The corresponding hourly changes in user welfare are illustrated in Figure 9a. The results indicate that the gas demand of residential users is relatively inelastic, and dynamic price adjustments do not significantly affect the basic living needs of residents. As a result, there may be a certain decline in user convenience, and the variation in user utility exhibits volatility in response to price adjustments. During peak periods, due to higher natural gas prices, the total user welfare decreases, whereas during low-price periods, user welfare increases. Overall, due to the significant reduction in gas purchase costs outweighing the loss in user utility, residential users benefit economically, thereby enhancing the overall level of gas consumption welfare.

The gas purchase costs, utility, and welfare for non-residential users are presented in Table 7. Overall, the decline in user utility alongside an increase in user welfare indicates that the savings in gas purchase costs significantly outweigh the loss in utility. As a result, the final user welfare has significantly improved, increasing by 2224.01 compared to the pre-optimization scenario. This trade-off between utility and cost is particularly pronounced among non-residential users. The corresponding hourly variation in user welfare is shown in Figure 9b. It can be observed that during valley periods, the welfare of non-residential gas users increases significantly, whereas it declines during flat and peak periods. However, since the magnitude of the increase during valley periods exceeds the decrease observed during flat and peak periods, the total welfare exhibits an upward trend over the scheduling cycle. The results indicate that, due to the higher gas consumption elasticity of non-residential users, adjusting production schedules in response to price signals during valley periods can significantly enhance user welfare.

It is noteworthy that the results in Table 6 and Table 7 show a much higher welfare increase for non-residential users (88.87%) compared with residential users (4.64%). This difference clearly reflects the gap in response capability and adjustment potential between residential and non-residential users under price signals. Residential users mainly use natural gas to meet basic daily needs, and their gas consumption behavior is relatively inelastic. Non-residential users possess greater scheduling flexibility over time, enabling them to shift energy-intensive processes to the valley period when gas prices are lower by adjusting production plans or process arrangements, thereby significantly reducing gas costs. Therefore, the reduction in gas costs for non-residential users is much greater than the slight decrease in their utility, resulting in a significant net welfare gain. These findings indicate that under price-based demand response mechanisms, consumers with greater temporal flexibility and load elasticity can achieve higher economic welfare gains through optimized gas usage scheduling and cost management.

The scheduling optimization not only effectively reduces the operating costs of gas suppliers but also significantly enhances user welfare, thereby achieving a win-win scenario for both gas suppliers and users. In addition to notable economic benefits, the dynamic pricing mechanism, when integrated with carbon emission constraints and carbon trading schemes, demonstrates differentiated environmental impacts across various user groups. This study proposes differentiated carbon emission management strategies for different types of users. Residential users, due to their relatively low gas consumption and non-participation in carbon trading, have their carbon emissions calculated and constraints set within the model. In contrast, non-residential users, characterized by higher gas consumption and the involvement of certain industrial users in carbon trading, require the incorporation of a ladder-type carbon trading cost structure. This strategy aligns with the practical conditions of future natural gas markets and provides theoretical support for the differentiated management of carbon emissions among various gas consumers.

**Figure 10:**
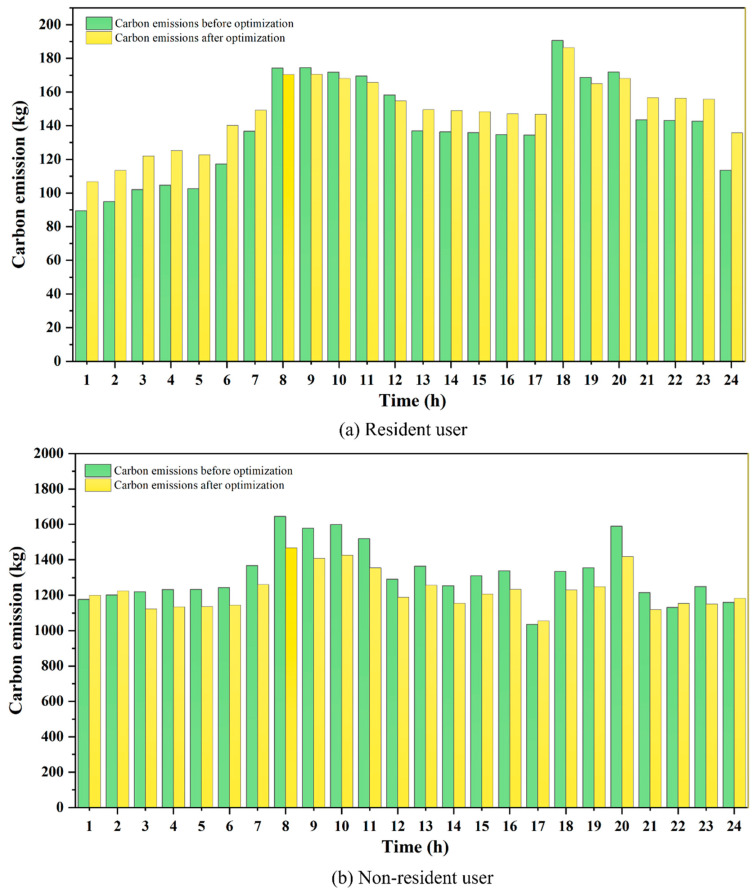
Carbon emissions before and after scheduling optimization.

Table 8 presents the changes in natural gas carbon emissions and carbon trading costs for residential and non-residential users before and after the scheduling optimization. The corresponding changes in hourly carbon emissions before and after optimization are shown in Figure 10. The results indicate that residential users have a relatively small baseline gas consumption and do not directly participate in carbon trading. Under dynamic pricing and carbon emission constraints, their emissions decrease from 3349.16 kg to 3233.98 kg, representing a reduction of 115.18 kg or approximately 3.44 percent. This suggests that through effective scheduling optimization, significant reductions in carbon emissions can be achieved for residential users. For non-residential users, the baseline gas consumption and associated emissions are significantly higher, and some industrial users bear tiered carbon trading costs. Under the combined effect of dynamic pricing and carbon trading, their carbon emissions decrease from 31,650.49 kg to 29,493.69 kg, representing a reduction of 2156.8 kg or about 6.82 percent. At the same time, the carbon trading cost declines from 2035.57 CNY to 1851.72 CNY, a reduction of 183.85 CNY. This indicates that the LTCTM not only contributes to reducing carbon emissions from non-residential users but also effectively controls their carbon trading costs, thereby managing the overall carbon emissions of the system. In summary, residential users, as low-demand consumers, primarily contribute to relative emission reduction and the stabilization of carbon emission fluctuations. Non-residential users, as high-demand consumers, play a leading role in total emission reduction and the decline of carbon trading costs. The differentiated responses of the two user groups enable the system to balance stability and aggregate emission reduction, thereby supporting the long-term reliable operation of natural gas scheduling.

### 4.3. Analysis of System Benefits Under Different Price Signal Guidance

To further examine the model’s sensitivity and robustness under different price elasticity settings, and to assess the role of time-of-use price elasticity in regulating natural gas prices, guiding user load reduction and shifting, and mitigating peak-valley load differences, this section designs several comparative scenarios with varying elasticity settings. As shown in Table 9, four scenarios are set, with RU_F and RU_C representing the scheduling scenarios resulting from changes in residential user gas demand due to price fluctuations under fixed price elasticity and time-of-use price elasticity. Similarly, NRU_F and NRU_C represent the scheduling scenarios after the variation in gas demand from non-residential users due to price changes under fixed price elasticity and time-of-use price elasticity. In this context, the fixed price elasticity for users is based on their own price elasticity, with residential users and non-residential users having values of −0.233 and −0.239 [38,40]. The time-of-use price elasticity follows the values presented in Table 3.

**Figure 11:**
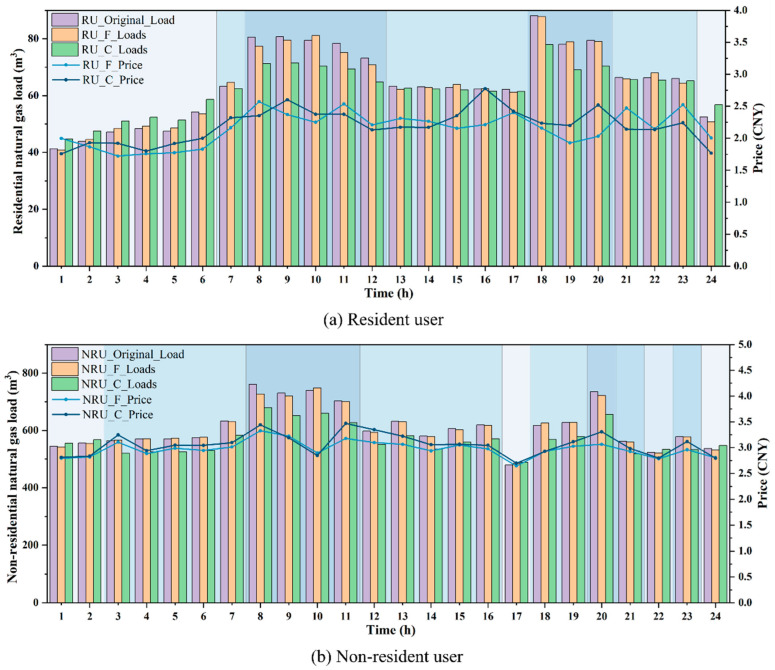
The load variation and dynamic pricing of users under different price elasticity mechanisms.

Figure 11 compares the load variations and dynamic price adjustments for residential and non-residential users, contrasting the effects of fixed price elasticity with those of time-of-use price elasticity. RU_Original_Load and NRU_Original_Load refer to the original natural gas load for residential users and non-residential users. It can be observed that although the RU_F scenario induces a slight increase in gas consumption during valley and flat periods, and a slight decrease during peak periods, the overall guiding effect is not significant. This is due to the inability of price adjustments to meet users’ psychological expectations, resulting in reverse fluctuations at specific time points (for example, a decrease in gas consumption at 1:00 and 24:00, and an increase in consumption at 10:00 and 19:00). The RU_C scenario can effectively guide users to increase their gas consumption during valley and flat periods, while reducing consumption during peak periods, thereby alleviating the scheduling pressure during peak times. In the NRU_F scenario, the guiding effect of the valley and flat periods is not significant, manifesting only as slight variations in gas consumption. During peak periods, gas consumption decreases, but at certain time points (e.g., 10:00), there is a slight increase in consumption, indicating that the regulatory effect of this scenario on user gas consumption behavior is not pronounced. In the NRU_C scenario, the gas consumption during valley hours significantly increases, while the gas consumption during flat and peak hours notably decreases. Therefore, the dynamic pricing determined by the fixed price elasticity has a limited effect on guiding users’ gas consumption behavior, and certain nodes are prone to fluctuations. The dynamic pricing determined by time-of-use price elasticity exhibits significant effectiveness in load management, effectively shifting and reducing natural gas load. Dynamic pricing, developed based on time-of-use price elasticity, can effectively shift and reduce natural gas load. It ensures safer and more stable scheduling during peak periods, thereby preventing the system from experiencing excessive pressure.

Table 10 and Table 11 present the economic benefits of the gas supplier and users during the scheduling process under different scenarios. For the gas supplier, the purchasing costs, distribution costs, and compressor operation costs in the RU_C scenario are lower than those in the RU_F scenario, while the revenue in the RU_C scenario is higher than that in the RU_F scenario. Similarly, in the gas scheduling for non-residential users, NRU_C also demonstrates lower costs and higher benefits. In terms of user benefits (as shown in Table 11), the user welfare in the RU_C scenario is slightly higher than that in the RU_F scenario, while the user welfare in the NRU_C scenario exceeds that in the NRU_F scenario by 772.67. The results indicate that, under the influence of the time-of-use price elasticity matrix, the cost for gas suppliers is reduced, and their revenue is enhanced. At the same time, the welfare of both residential and non-residential users is improved, thereby enabling the realization of a more economically efficient scheduling solution.

Additionally, Table 12 presents the carbon emissions, carbon trading costs, and load volatility of users under different scenarios. The carbon emissions in the RU_C scenario are 98.7 kg lower than those in the RU_F scenario. In the NRU_C scenario, carbon emissions are reduced by 1678.61 kg compared to the NRU_F scenario, resulting in a savings of 171.67 CNY in carbon trading costs. This indicates that the dynamic pricing strategy derived from time-of-use price elasticity outperforms the fixed price elasticity in terms of environmental benefits. Additionally, the load variation induced by the RU_C scenario results in a reduction in the volatility from the original value of 0.2059 to 0.1333. In contrast, the volatility for RU_F decreases only to 0.2006, indicating a relatively limited adjustment effect. The NRU_C scenario reduces the load volatility from the original value of 0.1221 to 0.0899. In contrast, the NRU_F scenario only adjusts it to 0.1166, with the adjustment effect being less pronounced. It is evident that time-of-use dynamic pricing performs better than fixed pricing in reducing load volatility and lowering carbon emissions. For both residential and non-residential gas scheduling, dynamic price signals derived from price elasticity are more effective than fixed-price elasticity in guiding gas consumption behavior and regulating gas volume, thereby mitigating load variability. In addition, the environmental benefits and load volatility effects of time-of-use dynamic pricing show heterogeneous patterns between residential and non-residential users. Non-residential users achieve greater emission reductions and carbon trading cost savings because of larger gas consumption, flexible production schedules, and stronger exposure to carbon cost constraints. In contrast, residential users exhibit more pronounced improvements in load smoothing, yet their emission reductions remain limited due to the rigidity of household energy demand. Therefore, the time-of-use dynamic pricing mechanism has potential to enhance system scheduling efficiency and to facilitate the implementation of energy-saving and emission-reduction policies.

### 4.4. Analysis of Load Guidance and User Utility Under Different Gas Consumption Behaviors

For gas suppliers, user gas consumption behavior directly affects load distribution as well as cost and revenue. A well-optimized gas procurement strategy can reduce procurement and transmission costs while maintaining the stability of gas distribution within the system. For end users, adjustments in gas consumption behavior influence gas usage costs and user welfare, contributing to the reduction in unnecessary waste. User gas consumption behavior is primarily influenced by user utility. Equation (11) incorporates price sensitivity and load variation acceptance into the utility function to characterize users’ responses to price signals and load adjustments. To analyze and verify the model’s sensitivity and user behavioral patterns under different combinations of $\lambda_{p}$ and $\lambda_{g}$, this section adopts the residential gas load and relevant parameters from Section 4.1 as fixed data. Four different parameter combinations representing gas consumption preferences are established, as shown in Table 13.

**Figure 12:**
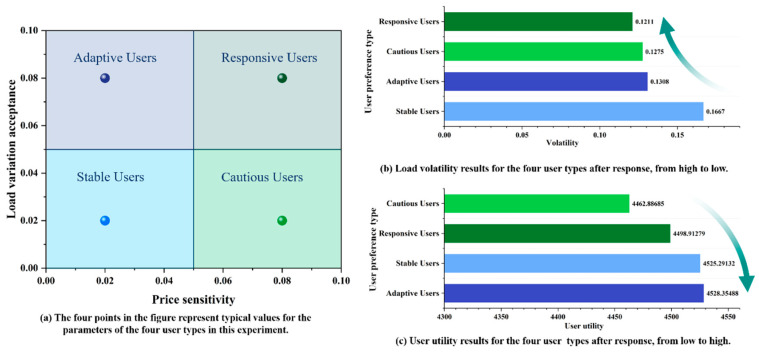
Typical parameter values for price sensitivity and load variation acceptance among users with different preference types, as well as the corresponding load volatility and user utility results after user response.

Figure 12a illustrates the interval settings of adaptive, stable, responsive, and cautious users in terms of price sensitivity and load variation acceptance. The median-proximal value within the interval is selected as the representative parameter. This approach preserves heterogeneity while avoiding extreme situations, thereby providing more representative input parameters for the simulation experiments. The [$\lambda_{p}$, $\lambda_{g}$] values for the four types are [0.02, 0.08] for adaptive users, [0.02, 0.02] for stable users, [0.08, 0.08] for responsive users, and [0.08, 0.02] for cautious users. Figure 12b,c present the load volatility and utility rankings of the four user categories after scheduling optimization. The results indicate clear differences in performance. Stable users exhibit the highest volatility (0.1667) with a moderate utility level (4525.29132). Responsive users achieve the lowest volatility (0.1211) while maintaining relatively high utility (4498.91279). Cautious users perform poorly in both volatility (0.1275) and utility (4462.88685). Adaptive users strike a balance between utility (4523.35488) and volatility (0.1308), reflecting a more resilient response pattern.

**Figure 13:**
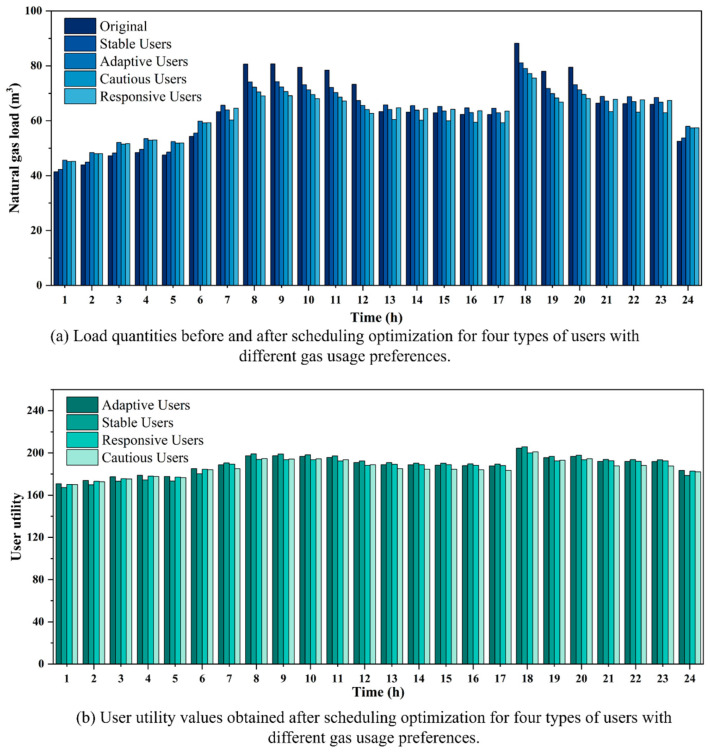
Hourly natural gas load and user utility under different user types after scheduling optimization.

On the one hand, guiding users to adjust their required load through price signals to achieve peak shaving and valley filling contributes to enhancing the overall stability and security of the scheduling system. From the perspective of peak shaving and valley filling capabilities, responsive users exhibit high sensitivity to price changes, demonstrating the strongest load adjustment ability. Under price guidance, it is capable of flexibly adjusting the load fluctuations within the scheduling period to adapt to price changes. These users exhibit high values of $\lambda_{p}$ and $\lambda_{g}$, enabling them to respond actively under dynamic pricing strategies, thereby providing significant flexibility for optimizing scheduling. Stable users, due to their insensitivity to price fluctuations, lack the motivation to adjust their load. Additionally, constrained by gas usage plans and practical conditions, their acceptance of load variations is relatively low. As a result, they tend to maintain a stable consumption pattern, demonstrating the weakest peak-shaving and valley-filling capabilities. The peak-shaving and valley-filling capacity of cautious users is second only to that of responsive users. Due to their higher value of $\lambda_{p}$, they exhibit a stronger willingness to adjust their load. However, due to the constraints imposed by inflexible loads, the load regulation capability of such users is limited, necessitating a reduction in utility to accommodate load variations. Therefore, a trade-off must be made between economic benefits and gas usage stability. Although adaptive users exhibit strong load regulation capability, their relatively low $\lambda_{p}$ results in limited motivation for voluntary load adjustment. Adjustments are made moderately, with consideration for economic benefits, only when significant price changes occur. The details of load fluctuation of users with different gas consumption preferences in a scheduling period are shown in Figure 13a.

On the other hand, when guiding users to adjust their gas consumption, their actual willingness must be considered. The utility values of the four user types are shown in Figure 12c. The results indicate the following ranking of user utility values: Adaptive Users > Stable Users > Responsive Users > Cautious Users. Adaptive users exhibit low price sensitivity and significant load flexibility, enabling rational load adjustment while maintaining comfort levels. These users experience optimal outcomes under stable and fluctuating prices, resulting in the highest utility values. Stable users exhibit a weak response to price and load fluctuations, with their gas consumption patterns minimally affected by price variations, maintaining a consistent demand. Although lacking regulation capacity, such users maintain high psychological utility due to stable gas consumption. The utility of responsive users is relatively low. Although they can actively respond to price adjustments and optimize costs through load regulation, their consumption patterns are significantly impacted, leading to additional adjustment costs and affecting comfort. As a result, their overall utility is lower than that of adaptive and stable users. Cautious users are sensitive to price changes, but due to their strong load rigidity, they are reluctant to accept significant load adjustments. These users accept load adjustments forcefully to reduce costs, sacrificing their own gas consumption experience, resulting in the lowest overall utility. The details of user utility with different gas consumption preferences in a scheduling period are shown in Figure 13b.

### 4.5. The Effect of Different Decision-Making Mechanism Factors on Scheduling Strategy Selection

In the EW-VIKOR method, the decision-making factor ν serves as a key parameter to represent preference in the evaluation process. It balances between minimizing the maximum regret, which reflects risk aversion, and maximizing the overall benefit, which reflects collective orientation. When ν takes a smaller value, the model places greater emphasis on reducing the worst performance of individual objectives, thereby indicating a conservative decision-making attitude. When ν takes a larger value, the model emphasizes the overall average level, reflecting a tendency toward risk preference or collective utility maximization. When ν equals 0.5, the system exhibits a risk-neutral attitude [41]. To evaluate the influence of ν on the final optimal trade-off solution, this section conducts a fine-grained analysis of ν within the range [0, 1] using a step size of 0.01. Sensitivity experiments are performed separately on the datasets of residential and non-residential users (as presented in Section 4.1). The analysis compares the variations and practical implications of supplier cost, user welfare, carbon emissions, and load volatility under different values of ν.

**Figure 14:**
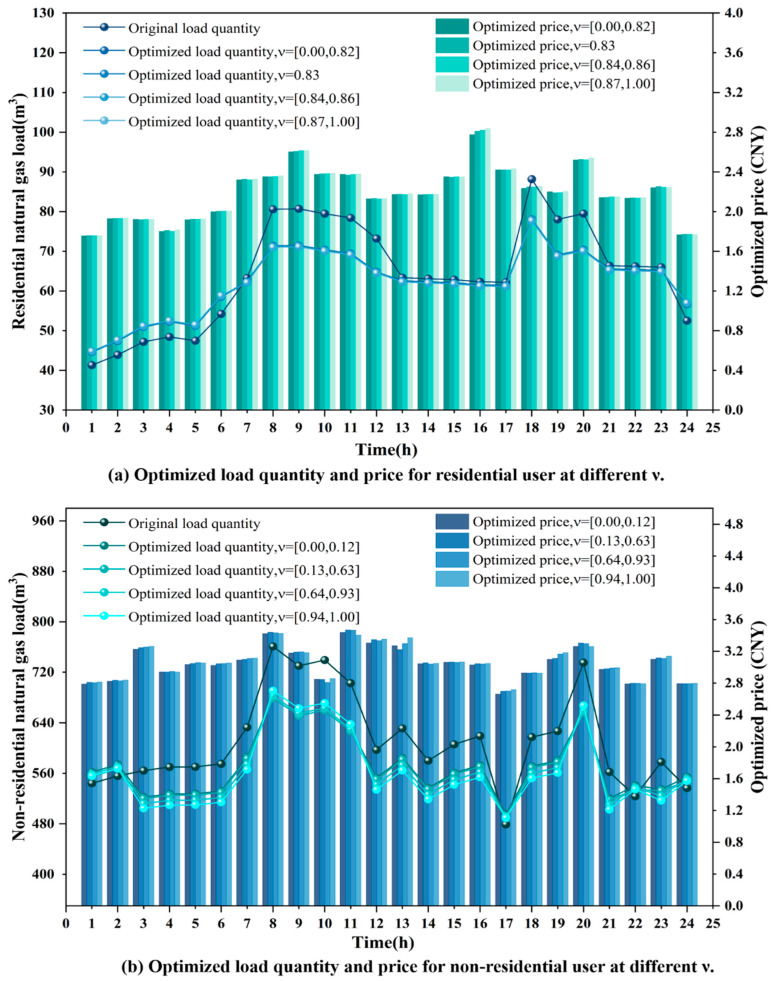
Results of residential and non-residential load optimization at different ν (in increments of 0.01).

Figure 14a,b present the optimized load and price results for residential and non-residential users under different values of ν. The results show that the load and price curves of residential users exhibit only minor variations across different decision-making factors ν. This indicates that their adjustment potential is limited, while the outcomes remain robust. Non-residential users show higher sensitivity to ν. A low ν value leads to significant peak load reduction and is suitable for scenarios that emphasize system stability and security. A high ν value results in weaker load reduction during certain peak periods, such as 10 h and 20 h, and focuses more on improving overall system efficiency. Also, the price level is higher in certain periods (such as 13 h, 19 h, and 23 h), which further encourages load shifting to other hours.

Table 14 and Table 15 indicate that variations in the decision-making factor ν under the EW-VIKOR method show clear differences between residential and non-residential users. For residential users, supplier cost, user welfare, carbon emissions, and volatility after demand response are all improved compared with the pre-response case. Within the interval [0.00, 0.82], the supplier cost decreases by 74.64 CNY, user welfare increases by 52.28, carbon emissions decrease by 115.18 kg, and volatility declines by 0.0726. When ν increases to 0.83 or higher, the variations in economic and environmental benefits become minor, reflected only in slight fluctuations in user welfare and carbon emissions. Overall, the system performance remains at a favorable level. This indicates that the model results are robust to changes in ν. Hence, for residential users, selecting a moderate decision-making factor, such as ν = 0.5, achieves a balanced outcome in both economic efficiency and environmental performance, without excessive reliance on ν adjustment.

Compared with residential users, non-residential users show more sensitive changes in economic and environmental benefits under different values of ν, which highlights distinct differences in decision-making preferences. When the value of ν is set within [0.00, 0.12], the system scheduling outcome shifts toward conservative objectives. The supplier cost decreases by 1745.56 CNY, while carbon emissions and carbon trading costs are reduced by 1979.9 kg and 170.47 CNY. The volatility declines by 0.0327. Meanwhile, user welfare significantly increases by 2236.06 under stable operating conditions. This outcome is suitable for scenarios where operational security and risk avoidance are prioritized. As ν increases to the ranges [0.13, 0.63] and [0.64, 0.93], the scheduling scheme shifts gradually toward overall benefit. Supplier costs and carbon emissions continue to decline, while user welfare rises further, reaching a peak of 4861.77. However, the volatility begins to increase. When ν approaches 1, the decision orientation clearly favors collective benefit maximization. The supplier cost reaches the minimum, user welfare attains the highest level, and both carbon emissions and carbon trading costs remain at their optimal values. However, the volatility decreases only by 0.016, indicating a limited improvement in system stability.

In summary, residential users achieve robust scheduling performance when the decision mechanism factor is set at the mid-range value (0.5). In contrast, the scheduling results of non-residential users are more sensitive to parameter settings. Strategies corresponding to smaller ν values favor system stability and fluctuation suppression. Strategies with larger ν values promote economic benefits and emission reduction targets. Therefore, applying the EW-VIKOR method to the Pareto solution set obtained from NSGA-III provides differentiated decision support for different user types and enables a flexible balance among economic performance, environmental outcomes, and system stability.

## 5. Conclusions

In the context of energy transition and increasingly stringent carbon emission constraints, natural gas scheduling optimization must balance economic efficiency, user behavior response, and low-carbon objectives. This study differs from conventional IES models that focus on high-level coordination between electricity and gas. Instead, it is the first to incorporate demand-side flexibility and carbon emission constraints into an independently scheduled, point-to-point NGS, enabling fine-grained control over downstream gas consumption. Meanwhile, traditional natural gas scheduling methods often overlook the complexity of user consumption behavior, limiting the effectiveness of price signals and lacking a systematic approach to differentiated carbon emission management for various types of users. To address these issues, this paper focuses on incorporating dynamic price signals and user behavior modeling, providing strategies for NGLCES that enhance economic efficiency, environmental sustainability, stability, and scalability. The model introduces a time-of-use price elasticity mechanism, leveraging price signals to guide gas consumption for different user types. Simultaneously, by incorporating user sensitivity to price changes and acceptance of load variations, a more accurate user behavior model is achieved, aligning the model more closely with actual user gas consumption considerations and habits. In carbon emission management, differentiated carbon emission constraints and targets are proposed for residential and non-residential users, effectively enhancing environmental awareness. Ultimately, the NSGA-III algorithm, assisted by MCDM, addresses high-dimensional MOPs and derives the optimal scheduling strategy. The comparative experiments focus on the benefits of price signal regulation and user behavior response, providing a scalable scheduling strategy for NGS applicable to users with different gas consumption preferences.

Validated with real data from a natural gas station branch in Xi’an, the simulation experiments confirm that the proposed model improves both the economic efficiency and environmental sustainability of scheduling. After optimization, the natural gas load of residential users decreased by 53.27 m^3^, with the load volatility reduced to 0.1333. For non-residential users, the load decreased by 997.51 m^3^, with the volatility reduced to 0.0899. These results indicate that the cross-price mechanism effectively smooths load peaks and valleys, enhances system stability, and validates the guiding role of dynamic price signals in load regulation. The NGLCES model optimizes gas supply and demand scheduling, significantly reducing supplier operating costs and enhancing user welfare. After scheduling optimization, the gas supplier’s costs decreased while user welfare improved. The total welfare of residential users increased by 52.28, and that of non-residential users rose by 2224.01, achieving a win-win outcome for both the gas supplier and users. In terms of environmental benefits, the optimized scheme reduces carbon emissions by 115.18 kg for residential users and 2156.8 kg for non-residential users, while lowering carbon trading costs by 183.85 CNY, confirming the effectiveness of the low-carbon-oriented scheduling strategy. Additionally, comparative experiments analyze the impact of different price signal guidance and gas usage behaviors on the optimized scheduling strategy. On the one hand, by analyzing system benefits under fixed and time-of-use price elasticity, it is verified that time-of-use price elasticity can guide users to make more realistic and beneficial load responses. On the other hand, based on price sensitivity and load variation acceptance, users are categorized into four types: adaptive, stable, responsive, and cautious, with their load fluctuations and psychological utility analyzed separately. The results indicate that responsive users exhibit the lowest load fluctuation rate after scheduling optimization, actively adjusting gas consumption based on price signals. Adaptive users achieve the highest utility and exhibit the greatest satisfaction with scheduling optimization results.

The proposed model serves as a decision support tool for natural gas dispatch centers to generate optimized real-time pricing schemes. By inputting user load data and modeling parameters, the model derives pricing strategies that balance economic efficiency and environmental sustainability, considering supplier costs, user welfare, load volatility, and carbon emissions. For dispatch centers, the model provides quantitative guidance for designing flexible scheduling and pricing plans. Gas suppliers can optimize supply schedules based on the model outputs to reduce costs and maintain system stability. Users can adjust production or gas consumption in response to real-time price signals to save costs and reduce emissions. Policymakers and regulators can use the model to assess the system impacts of different carbon trading policies or pricing mechanisms, supporting the integration of long-term carbon trading mechanisms with dispatch strategies. The proposed model demonstrates strong transferability despite being primarily validated using data from Xi’an, a northern Chinese city. Future studies should extend the analysis to datasets from different regions and seasons to evaluate the model’s generalizability and robustness. Considering extreme events and socioeconomic variations can further enhance its practical adaptability and reliability. In addition, carbon emission management remains a promising area for expansion. Incorporating the effects of regional carbon market regulations and policy changes can strengthen the flexibility and applicability of emission management strategies.

## Figures and Tables

**Table 1:** Relevant parameters.

Parameters	Residential Users	Non-Residential Users
χ	0.8	0.8
$\mu (kg\;{CO}_{2}/m^{3})$	2.1622	2.1622
λ	/	0.252
ω	/	25%
l	/	2000
$\lambda_{p}$	[0, 0.1]	[0, 0.1]
$\lambda_{g}$	[0, 0.1]	[0, 0.1]

Notes: “/” denotes that the indicator or variable is not required to be calculated in that state.

**Table 2:** Price related parameters.

Price (CNY)	Residential Users	Non-Residential Users
$P_{0}$	2.18	3.04
$P_{up}$	3.27	3.648
$P_{pl}$	1.23	1.403
$P_{sto}$	1.23	1.403
$P_{lng}$	3.31	5.875
$P_{ptp}$	0.465	0.48

**Table 3:** Price elasticity matrix.

User Type	Period	Peak	Flat	Valley
Residential Users	Peak	−0.233	0.0621	0.0466
	Flat	0.0621	−0.233	0.0311
	Valley	0.0466	0.0311	−0.233
Non-residential Users	Peak	−0.239	0.04	0.03
	Flat	0.04	−0.239	0.02
	Valley	0.03	0.02	−0.239

**Table 4:** Costs, revenues, and profits of gas suppliers (Scheduling for residential users).

State	Gas Supplier Cost (CNY)			Total Supplier Cost (CNY)	Gas Supplier Revenue (CNY)	Gas Supplier Profit (CNY)
	Purchase Cost	Distribution Cost	Compressor Operating Cost			
Before scheduling optimization	1905.22	216.85	36.03	2158.10	3376.73	1218.63
After scheduling optimization	1839.70	209.40	34.36	2083.46	3313.58	1230.12
Difference	−65.52	−7.45	−1.67	−74.64	−63.15	11.49

**Table 5:** Costs, revenues, and profits of gas suppliers (Scheduling for non-residential users).

State	Gas Supplier Cost (CNY)			Total Supplier Cost (CNY)	Gas Supplier Revenue (CNY)	Gas Supplier Profit (CNY)
	Purchase Cost	Distribution Cost	Compressor Operating Cost			
Before scheduling optimization	20,537.25	7026.29	320.99	27,884.53	44,499.82	16,615.29
After scheduling optimization	19,137.75	6547.48	297.91	25,983.14	41,852.11	15,868.97
Difference	−1399.50	−478.81	−23.08	−1901.39	−2647.71	−746.32

**Table 6:** Gas purchase costs, utility, and welfare of residential users.

State	Gas Purchase Cost (CNY)	User Utility	User Welfare
Before scheduling optimization	3376.73	4502.32	1125.59
After scheduling optimization	3313.58	4491.45	1177.87
Difference	−63.15	−10.87	52.28

**Table 7:** Gas purchase costs, utility, and welfare of non-residential users.

State	Gas Purchase Cost (CNY)	User Utility	User Welfare
Before scheduling optimization	44,499.82	47,002.16	2502.34
After scheduling optimization	41,852.11	46,578.46	4726.35
Difference	−2647.71	−423.7	2224.01

**Table 8:** Carbon emissions and carbon trading costs for different users.

User Type	State	Carbon Emissions (kg)	Carbon Trading Costs (CNY)
Residential Users	Before scheduling optimization	3349.16	/
	After scheduling optimization	3233.98	/
	Difference	−115.18	/
Non-residential Users	Before scheduling optimization	31,650.49	2035.57
	After scheduling optimization	29,493.69	1851.72
	Difference	−2156.8	−183.85

Notes: “/” denotes that the indicator or variable is not required to be calculated in that state.

**Table 9:** Scenario division of residential and non-residential users under different price signals.

Scenario Id	User Type	Fixed Price Elasticity	Time-of-Use Price Elasticity	Characteristics
RU_F	Resident users	√		Unified elasticity parameter for all time periods.
RU_C	Resident users		√	Differentiated elasticity parameters for peak, flat, and valley periods.
NRU_F	Non-resident users	√		Unified elasticity parameter for all time periods.
NRU_C	Non-resident users		√	Differentiated elasticity parameters for peak, flat, and valley periods.

Notes: “√” indicates the selected user type and price elasticity type in the corresponding scenario.

**Table 10:** The costs, revenues, and profits of the gas supplier under different scenarios.

Scenario Id	Gas supplier Cost (CNY)			Total Supplier Cost (CNY)	Gas Supplier Revenue (CNY)	Gas Supplier Profit (CNY)
	Purchase Cost	Distribution Cost	Compressor Operating Cost			
RU_F	1895.84	215.79	35.81	2147.44	3360.01	1212.57
RU_C	1839.70	209.40	34.36	2083.46	3313.58	1230.12
NRU_F	20,447.64	6995.63	319.29	27,762.56	43,537.05	15,774.49
NRU_C	19,137.75	6547.48	297.91	25,983.14	41,852.11	15,868.97

**Table 11:** The gas purchasing costs, utility, and welfare of the users under different scenarios.

Scenario Id	Gas Purchase Cost (CNY)	User Utility	User Welfare
RU_F	3360.01	4534.89	1174.89
RU_C	3313.58	4491.45	1177.87
NRU_F	43,537.05	47,490.73	3953.68
NRU_C	41,852.11	46,578.46	4726.35

**Table 12:** The carbon emissions, carbon trading costs, and volatility of the users under different scenarios.

Scenario Id	Carbon Emissions (kg)	Carbon Trading Costs (CNY)	Load Volatility
RU_F	3332.68	/	0.2006
RU_C	3233.98	/	0.1333
NRU_F	31,172.3	2023.39	0.1166
NRU_C	29,493.69	1851.72	0.0899

Notes: “/” denotes that the indicator or variable is not required to be calculated in that scenario.

**Table 13:** The four parameter combinations of gas consumption preferences and behavioral characteristics.

Preference Type	$\lambda_{p}$	$\lambda_{g}$	Parametric Characteristics
Adaptive Users	(0, 0.05]	(0.05, 0.1]	Low price sensitivity + High load variation acceptance
Stable Users	(0, 0.05]	(0, 0.05]	Low price sensitivity + Low load variation acceptance
Responsive Users	(0.05, 0.1]	(0.05, 0.1]	High price sensitivity + High load variation acceptance
Cautious Users	(0.05, 0.1]	(0, 0.05]	High price sensitivity + Low load variation acceptance

**Table 14:** The economic and environmental benefit values of residential users under different ν intervals with a step size of 0.01.

ν	Gas Supplier Cost (CNY)	User Welfare	Carbon Emissions (kg)	Load Volatility
Before scheduling optimization	2158.10	1125.59	3349.16	0.2059
[0.00, 0.82]	2083.46	1177.87	3233.98	0.1333
0.83	2079.56	1175.44	3227.90	0.1346
[0.84, 0.86]	2080.73	1173.60	3229.74	0.1321
[0.87, 1.00]	2077.87	1169.03	3225.31	0.1310

**Table 15:** The economic and environmental benefit values of non-residential users under different *ν* intervals with a step size of 0.01.

ν	Gas Supplier Cost (CNY)	User Welfare	Carbon Emissions (kg)	Carbon Trading Costs (CNY)	Load Volatility
Before scheduling optimization	27,884.53	2502.34	31,650.49	2035.57	0.1221
[0.00, 0.12]	26,138.97	4738.40	29,670.59	1865.10	0.0894
[0.13, 0.63]	25,983.14	4726.35	29,493.69	1851.72	0.0899
[0.64, 0.93]	25,827.81	4861.77	29,317.03	1838.37	0.0957
[0.94, 1.00]	25,670.14	4962.44	29,137.49	1824.79	0.1061

## Data Availability

Data of this study are available on request from the corresponding author.

## Supplementary Materials

The following supporting information can be downloaded at: https://www.mdpi.com/article/10.3390/e27111120/s1.

## Abbreviations

The following abbreviations are used in this manuscript:

Nomenclature	
Abbreviations	
LNG	Liquefied natural gas
CNG	Compressed natural gas
DR	Demand response
DSM	Demand-side management
NGPN	Natural gas pipeline networks
NGS	Natural gas systems
LTCTM	Ladder-type carbon trading mechanism
NSGA-III	Non-dominated sorting genetic algorithm III
NGLCES	Natural gas low-carbon economic scheduling
CNY	Chinese yuan
DTW	Dynamic time warping
EW	Entropy weight method
VIKOR	VIseKriterijumska optimizacija i kompromisno resenje
MCDM	Multi-criteria decision-making
MOPs	Multi-objective optimization Problems
TOPSIS	Technique for order preference by similarity to an ideal solution
AHP	Analytic hierarchy process
CO_2_	Carbon dioxide
IES	Integrated energy system
Units	
CNY	Chinese yuan
kg	Kilogram
h	Hour
m^3^	Cubic meter
kg CO_2_/m^3^	Kilograms of CO_2_ per cubic meter (CO_2_ emission intensity)
Variables	
$∆g_{i}$	Change in natural gas demand at time i
$g_{i}^{0}$	Initial load at time i
$∆p_{i}$	Change in natural gas price at time i
$p_{i}^{0}$	Initial natural gas price at time i
$∆p_{j}$	Change in natural gas price at time j
$p_{j}^{0}$	Initial natural gas price at time j
$G_{f}$, $G_{p}$, $G_{g}$	Natural gas loads after DR for the peak, flat, and valley periods
$G_{f}^{0}$, $G_{p}^{0}$, $G_{g}^{0}$	Initial natural gas loads for the peak, flat, and valley periods
∆Q	The share of participation in carbon trading
$Q_{a}$	Actual natural gas carbon emissions
$Q_{quota}$	Carbon emission quota
*T*	The duration of a complete scheduling period
$C_{T}$	Costs of LTCTM
$∆P_{t}$	Normalized value of the change in price at time t
$∆G_{t}$	Normalized value of the change in load at time t
a, b	Adjusted benefit parameters
$P_{t}^{0}$	The initial price at time t
$P_{t}^{after}$	The price at time t after the scheduling optimization
$G_{t}^{0}$	The initial load at time t
$G_{t}^{after}$	The load at time t after the scheduling optimization
$U(G_{t})$	Improved utility function
$W(G_{t})$	User welfare function
$C_{pl}$	Procurement costs of pipeline gas
$C_{sto}$	Procurement costs of gas storage
$C_{lng}$	Procurement costs of LNG
$G_{pl,\;t}$	Quantities of gas procured from pipeline gas at time t
$G_{sto,\;t}$	Quantities of gas procured from gas storage at time t
$G_{lng,\;t}$	Quantities of gas procured from LNG at time t
$C_{ptp}$	Pipeline distribution costs
$G_{t}$	Natural gas load at time t
$C_{comp}$	Compressor operation cost
$W_{p,n}$	Power of the n-th compressor
$V_{load}$	Load fluctuation
$V_{eload}$	Load fluctuation difference
$E_{T}$	Total carbon emissions
$\bar{G}$	Average natural gas load over a scheduling period
$C_{cost}$	Gas supplier costs
$\bar{G^{0}}$	Average initial gas load
$G^{min}$,$G^{max}$	The upper and lower limits of load after response
$P_{g},\;P_{p}$, $P_{f}$	The prices during valley, flat, and peak periods
$V_{load}^{0}$	Load fluctuation before DR
$C_{cost}^{0}$	Gas supplier costs before DR
$S_{bp,t}$	Opening status indicators for the bypass valve at time t
${U(G_{t})}_{base}$	Basic utility function
$G_{bp,t}$	The natural gas flow rates through the bypass valve at time t
$G_{com,t}$	The natural gas flow rates through the compressor valve at time t
$S_{com,t}$	Opening status indicators for the compressor valve at time t
$G_{{it}_{1}}$	The natural gas load at time $t_{1}$ on the i-th day.
$G_{{jt}_{2}}$	The natural gas load at time $t_{2}$ on the j-th day.
$r_{a,b}$	The normalized value of the b-th objective for the a-th solution.
$f_{b}^{max}$,$f_{b}^{min}$	The maximum and minimum values of the b-th objective function.
$f_{a,b}$	The value of the b-th objective for the a-th solution.
$p_{a,b}$	The weight of the b-th objective for the a-th solution.
$E_{b}$	The entropy value of the b-th objective.
$w_{b}$	The weight assigned to the b-th objective.
$S_{a}$	The group utility of the a-th solution.
$R_{a}$	The individual regret of the a-th solution.
$f_{b}^{+}$*,*$f_{b}^{-}$	The positive and negative ideal solution for the b-th objective.
$Q_{a}$	The compromise value of the a-th solution.
$S^{+}$*,*$S^{-}$	The maximum and minimum values of group utility.
$R^{+}$*,*$R^{-}$	The maximum and minimum values of individual regret.
Parameters	
$\varepsilon_{ii}$	Own price elasticity coefficient at time i
$\varepsilon_{ij}$	Cross-price elasticity coefficient at two different times i and j
$E_{ij}$	Price elasticity matrix
$E_{ts}$	Price elasticity matrix after dividing peak, flat and valley
$\varepsilon_{ff}$,$\varepsilon_{pp}$,$\varepsilon_{gg}$	Self-elasticity coefficients for the peak, flat, and valley periods
$\varepsilon_{fp}$,$\varepsilon_{fg}$,$\varepsilon_{pg}$	Cross-elasticity coefficients between the peak, flat, and valley periods
χ	Carbon emission allocation coefficient
μ	Natural gas emission factor
λ	Benchmark price for carbon trading
ω	Growth rate of the price
l	The interval length
$a_{base}$,$b_{base}$	The base benefit parameters
c	Offset constant
$\lambda_{p}$,$\lambda_{g}$	Sensitivity coefficients for price and load variations
$P_{pl}$	Price of pipeline gas
$P_{sto}$	Price of gas storage
$P_{lng}$	Price of LNG
$P_{ptp}$	Transportation cost
L	Transportation distance
$t_{period}$	Operational duration of the n-th compressor
$p_{ce}$	The energy cost coefficient of the compressor
$n_{c}$	The number of compressors in operation
$P_{0}$	Initial gas price
$P_{up}$	Gas price ceiling
v	Trade-off factor
