# Supplementary material for: Low-Carbon Economic Operation of Natural Gas Demand Side Integrating Dynamic Pricing Signals and User Behavior Modeling
Source: Entropy (Basel). 2025 Oct 30;27(11):1120. doi: 10.3390/e27111120 (PMC12651277; doi:10.3390/e27111120)
Supplement: Supplementary file 1 [file entropy-27-01120-s001.zip › entropy-3921697-supplementary.pdf]

Low-carbon economic operation of natural gas demand side integrating dynamic pricing signals and user behavior modeling

Supplementary Materials

S1. Supplementary information for the compressor model

This paper begins with the compressibility of gas, sequentially calculates the head of the compressor, and ultimately derives the compressor's power. The specific formula is presented in Eqs.(S.1)-(S.3).

The compressibility factor is a critical parameter in describing the compressibility of natural gas. To simplify the calculations, this section employs the AGA formula to determine the compressibility factor [1]. The compressor head (H_{com}) is a critical energy metric representing compressor performance. It refers to the energy required to raise natural gas from inlet pressure (p_{in}) to outlet pressure (p_{out}) under specific operating conditions. This parameter serves not only as a core intermediate variable in compressor power calculations but also as a key indicator for evaluating operational efficiency and energy costs. Specifically, the actual input power (W_p) required for the compressor to complete the gas compression task is calculated based on the compressor head. By accurately assessing the compressor head and input power, scientific guidance can be provided for optimizing compressor operations, thereby effectively reducing system energy consumption and enhancing the economic and sustainable performance of natural gas transportation. The detailed compressor model and parameters are provided in Table S1.

$$Z = 1 + 0.257\frac{p_{avg}}{p_{cr}} - 0.533\frac{p_{avg}T_{cr}}{p_{cr}T_{avgr}} \tag{S.1}$$

$$H_{com} = ZRT_{in}\frac{\sigma}{\sigma-1}\left[\left(\frac{p_{out}}{p_{in}}\right)^{\frac{\sigma}{\sigma-1}} - 1\right] \tag{S.2}$$

$$W_p = 3600\frac{\rho G_{com}H_{com}}{\eta_{com}} \tag{S.3}$$

Table S1 The parameters of the compressor model.

Parameters of the compressor model	Abbreviations	Value
Inlet pressure (MPa)	p_{in}	1.8
Outlet pressure (MPa)	p_{out}	3.9
Inlet temperature (K)	T_{in}	290
Peak compressor efficiency	η_{com}	0.80
Off-peak compressor efficiency		0.85
Peak energy price (CNY/kWh)	p_{ce}	1.62
Off-peak energy prices (CNY/kWh)		1.26
Gas constant (KJ/kg·K)	R	0.518
Average volumetric polytropic index	σ	1.3
Natural gas compression factor	Z	0.854
Compressor inlet gas density (kg/m³)	ρ	0.746
Number of main compressors	/	1
Number of spare compressors	/	1

S2. Nomenclature

Nomenclature	
**Abbreviations**	
LNG	Liquefied natural gas
CNG	Compressed natural gas
DR	Demand response
DSM	Demand-side management
NGPN	Natural gas pipeline networks
NGS	Natural gas systems
DSM	Demand-side management
LTCTM	Ladder-type carbon trading mechanism
NSGA-III	Non-dominated sorting genetic algorithm III
NGLCES	Natural gas low-carbon economic scheduling
CNY	Chinese yuan
DTW	Dynamic time warping
EW	Entropy weight method
VIKOR	VIseKriterijumska optimizacija i kompromisno resenje
MCDM	Multi-criteria decision-making
MOPs	Multi-objective optimization Problems
TOPSIS	Technique for order preference by similarity to an ideal solution
AHP	Analytic hierarchy process
IES	Integrated energy system
**Variables**	
Δg_i	Change in natural gas demand at time i
g_i^0	Initial load at time i
Δp_i	Change in natural gas price at time i
p_i^0	Initial natural gas price at time i
Δp_j	Change in natural gas price at time j
p_j^0	Initial natural gas price at time j
G_f, G_p, G_g	Natural gas loads after DR for the peak, flat, and valley periods
G_f^0, G_p^0, G_g^0	Initial natural gas loads for the peak, flat, and valley periods
ΔQ	The share of participation in carbon trading
Q_a	Actual natural gas carbon emissions
Q_{quota}	Carbon emission quota
T	The duration of a complete scheduling period
C_T	Costs of LTCTM
ΔP_t	Normalized value of the change in price at time t
ΔG_t	Normalized value of the change in load at time t
a, b	Adjusted benefit parameters
P_t^0	The initial price at time t
P_t^{after}	The price at time t after the scheduling optimization
G_t^0	The initial load at time t

G_t^{after}	The load at time t after the scheduling optimization
$U(G_t)$	Improved utility function
$W(G_t)$	User welfare function
C_{pl}	Procurement costs of pipeline gas
C_{sto}	Procurement costs of gas storage
C_{lng}	Procurement costs of LNG
$G_{pl,t}$	Quantities of gas procured from pipeline gas at time t
$G_{sto,t}$	Quantities of gas procured from gas storage at time t
$G_{lng,t}$	Quantities of gas procured from LNG at time t
C_{ptp}	Pipeline distribution costs
G_t	Natural gas load at time t
C_{comp}	Compressor operation cost
$W_{p,n}$	Power of the n-th compressor
V_{load}	Load fluctuation
V_{eload}	Load fluctuation difference
E_T	Total carbon emissions
$\bar{G}$	Average natural gas load over a scheduling period
C_{cost}	Gas supplier costs
$\overline{G^0}$	Average initial gas load
G^{min},G^{max}	The upper and lower limits of load after response
P_g, P_p, P_f	The prices during valley, flat, and peak periods
V_{load}^0	Load fluctuation before DR
C_{cost}^0	Gas supplier costs before DR
$S_{bp,t}$	Opening status indicators for the bypass valve at time t
$U(G_t)_{base}$	Basic utility function
$G_{bp,t}$	The natural gas flow rates through the bypass valve at time t
$G_{com,t}$	The natural gas flow rates through the compressor valve at time t
$S_{com,t}$	Opening status indicators for the compressor valve at time t
G_{it_1}	The natural gas load at time t_1 on the i-th day.
G_{jt_2}	The natural gas load at time t_2 on the j-th day.
$r_{a,b}$	The normalized value of the b-th objective for the$a$-th solution.
f_b^{max},f_b^{min}	The maximum and minimum values of the b-th objective function.
$f_{a,b}$	The value of the b-th objective for the$a$-th solution.
$p_{a,b}$	The weight of the b-th objective for the a-th solution.
E_b	The entropy value of the b-th objective.
w_b	The weight assigned to the b-th objective.
S_a	The group utility of the a-th solution.
R_a	The individual regret of the a-th solution.
f_b^+,f_b^-	The positive and negative ideal solution for the b-th objective.
Q_a	The compromise value of the a-th solution.
S^+,S^-	The maximum and minimum values of group utility.
R^+,R^-	The maximum and minimum values of individual regret.
**Parameters**	

ε_{ii}	Self-price elasticity coefficient at time i
ε_{ij}	Cross-price elasticity coefficient at two different times i and j
E_{ij}	Price elasticity matrix
E_{ts}	Price elasticity matrix after dividing peak, flat and valley
ε_{ff},ε_{pp},ε_{gg}	Self-elasticity coefficients for the peak, flat, and valley periods
ε_{fp},ε_{fg},ε_{pg}	Cross-elasticity coefficients between the peak, flat, and valley periods
χ	Carbon emission allocation coefficient
μ	Natural gas emission factor
λ	Benchmark price for carbon trading
ω	Growth rate of the price
l	The interval length
a_{base},b_{base}	The base benefit parameters
c	Offset constant
λ_p,λ_g	Sensitivity coefficients for price and load variations
P_{pl}	Price of pipeline gas
P_{sto}	Price of gas storage
P_{lng}	Price of LNG
P_{ptp}	Transportation cost
L	Transportation distance
t_{period}	Operational duration of the n-th compressor
p_{ce}	The energy cost coefficient of the compressor
n_c	The number of compressors in operation
P_0	Initial gas price
P_{up}	Gas price ceiling
v	Trade-off factor

S3. The steps of the EW-VIKOR method

(1) The objective values of the obtained Pareto solution set are normalized to construct matrix $R = [r_{a,b}]_{m\times n}$. The normalization formulas for cost-type and benefit-type objective functions are given in Eqs. (S.4) and (S.5).

$$r_{a,b} = \frac{f_b^{max} - f_{a,b}}{f_b^{max} - f_b^{min}} \ (b \in C_{cost}, E_T/C_T, V_{eload}) \tag{S.4}$$

$$r_{a,b} = \frac{f_{a,b} - f_b^{min}}{f_b^{max} - f_b^{min}} \ (b \in U(G_t)) \tag{S.5}$$

Here, $r_{a,b}$ represents the normalized value of the b-th objective for the a-th solution. f_b^{max} and f_b^{min} denote the maximum and minimum values of the b-th objective, respectively. $f_{a,b}$ refers to the b-th objective value of the a-th solution.

(2) Establish the entropy weight model. First, compute the proportion $p_{a,b}$ of the b-th objective for the a-th solution (Eq. (S.6)). Then, calculate the entropy value E_b of the b-th objective based on the weights (Eq. (S.7)).

$$p_{a,b} = \frac{r_{a,b}}{\sum_{a=1}^{m} r_{a,b}} \tag{S.6}$$

$$E_b = -\frac{1}{ln(m)} \sum_{a=1}^{m} p_{a,b} \, ln \, p_{a,b} \tag{S.7}$$

(3) Assign weights to each objective.

$$w_b = \frac{1 - E_b}{\sum_{b=1}^{n} (1 - E_b)} \tag{S.8}$$

In Eq. (S.8), w_b represents the weight of the b-th objective.

(4) Calculate the group utility S_a and individual regret R_a of the pareto solution set.

$$S_a = \sum_{b=1}^{n} w_b \frac{f_b^+ - r_{a,b}}{f_b^+ - f_b^-} \tag{S.9}$$

$$R_a = \max_b \left(w_b \frac{f_b^+ - r_{a,b}}{f_b^+ - f_b^-} \right) \tag{S.10}$$

In Eqs (S.9)-(S.10), f_b^+ represents the positive ideal solution, where $f_b^+ = \max_b \{r_{a,b}\}$, and f_b^- represents the negative ideal solution, where $f_b^- = \min_b \{r_{a,b}\}$.

(5) Calculate the trade-off value Q_a of the pareto solution set (multiple

strategies).

$$Q_a = \nu \frac{S_a - S^+}{S^- - S^+} + (1-\nu)\frac{R_a - R^+}{R^- - R^+} \tag{S.11}$$

In Eq. (S.11), $S^+ = \underset{a}{max}\{S_a\}$, $S^- = \underset{a}{min}\{S_a\}$, $R^+ = \underset{a}{max}\{R_a\}$, $R^- = \underset{a}{min}\{R_a\}$. ν is the decision-making mechanism factor, where $\nu \in [0,1]$. When $\nu > 0.5$, the system emphasizes collective benefits and multiple objectives, reflecting a risk-seeking decision. When $\nu < 0.5$, the system focuses on the single worst-performing indicator, representing a risk-averse decision. When $\nu = 0.5$, both collective utility and individual regret are considered, indicating a risk-neutral decision [2].

S4. NSGA-III parameter configuration

The non-dominated sorting genetic algorithm III (NSGA-III) is employed to solve the multi-objective scheduling optimization model, and the algorithm is implemented on the MATLAB R2023b platform. To balance solution diversity and convergence performance, the population size is set to 100, and the maximum number of generations is 500. The crossover and mutation probabilities are set to 0.9 and 0.1, respectively, following commonly adopted parameter settings in multi-objective evolutionary algorithms. The algorithm terminates when the maximum number of iterations is reached. To enhance the stability and robustness of the results, multiple independent runs are conducted, and the Pareto-optimal front with the best performance and uniform distribution is selected as the final outcome.

SI References

[1] Zhou J, Qin C, Fu T, Liu S, Liang G, Li C, et al. Automatic response framework for large complex natural gas pipeline operation optimization based on data-mechanism hybrid-driven. Energ 2024;307:132610. https://doi.org/10.1016/j.energy.2024.132610.

[2] Gao F, Gao J, Huang N, Wu H. Selection of an economics-energy-environment scheduling strategy for a community virtual power plant considering decision-makers' risk attitudes based on improved information gap decision theory. Energ 2024;299:131401. https://doi.org/10.1016/j.energy.2024.131401.
